# Supplementary material for: A parametric bootstrap approach for computing confidence intervals for genetic correlations with application to genetically determined protein-protein networks
Source: HGG Adv. 2024 May 8;5(3):100304. doi: 10.1016/j.xhgg.2024.100304 (PMC11140211; doi:10.1016/j.xhgg.2024.100304)
Supplement: Document S1. Figures S1 and S2 [file mmc1.pdf]

HGGA, Volume *5*

Supplemental information

A parametric bootstrap approach for computing confidence intervals for genetic correlations with application to genetically determined protein-protein networks

Yi-Ting Tsai, Yana Hrytsenko, Michael Elgart, Usman A. Tahir, Zsu-Zsu Chen, James G. Wilson, Robert E. Gerszten, and Tamar Sofer

A parametric bootstrap approach for computing confidence intervals for genetic correlations with application to genetically-determined protein-protein networks: Supplementary Note

Decomposition of the total phenotypic variance into genetic and residual variance. 1
Derivation of the two outcomes covariance model. 2

Supplementary Note

Decomposition of the total phenotypic variance into genetic and residual variance.

Suppose that the linear model relating the outcome vector $\boldsymbol{y}$ (of length $n \times 1$) to covariates $\boldsymbol{X}$ and genetic effects $\boldsymbol{G}$ is given by

$$\boldsymbol{y} = \boldsymbol{X\beta} + \boldsymbol{G\alpha} + \boldsymbol{e},$$

Where $\boldsymbol{e}$ is a vector of errors independent of the genetic effects. Conditional on covariates $\boldsymbol{X}$, the covariance matrix of $\boldsymbol{y}$ can be written as:

$$cov(\boldsymbol{y} - \boldsymbol{X\beta}, \boldsymbol{y} - \boldsymbol{X\beta}) = cov(\boldsymbol{G\alpha} + \boldsymbol{e}, \boldsymbol{G\alpha} + \boldsymbol{e}) = cov(\boldsymbol{G\alpha}) + cov(\boldsymbol{e})$$

Where $cov(\boldsymbol{e}) = \sigma_e^2 \boldsymbol{I}_{n\times n}$, such that the errors are assumed independent across individuals.

To consider the genetic covariance between individuals, first let's assume that there are d variants modelled, and under approximating assumptions of independence of genetic variants, standardized to have mean 0 and variance 1. Then, for individual i:

$$cov(\boldsymbol{g}_i^T \boldsymbol{\alpha}) = var(\boldsymbol{g}_i^T \boldsymbol{\alpha}) = var(g_{i1}\alpha_1 + \cdots + g_{id}\alpha_d) =$$

$$= var(g_{i1})\alpha_1^2 + \cdots + var(g_{id})\alpha_d^2$$

$$= \sum_{j=1}^{d} \alpha_j^2 = \sigma_k^2$$

Where σ_k^2 is the genetic variance.

To model the covariance between the outcome of the i and j individuals, we rely on the over-all probability (i.e., across the genome) that a variant is identical-by-descent between the two individuals. For this we use the kinship coefficient k_{ij}, and have:

$$cov\left(\boldsymbol{g}_i^T \boldsymbol{\alpha}, \boldsymbol{g}_j^T \boldsymbol{\alpha}\right) = cov\left(g_{i1}\alpha_1 + \cdots + g_{id}\alpha_d, g_{j1}\alpha_1 + \cdots + g_{jd}\alpha_d\right) =$$

$$= cov(g_{i1}, g_{j1})\alpha_1^2 + \cdots + cov(g_{id}, g_{jd})\alpha_d^2$$
$$= k_{ij}\sum_{j=1}^{d}\alpha_j^2 = k_{ij}\sigma_k^2$$

Note here that

$$\sum_{j=1}^{d}\alpha_j^2 = \sigma_k^2$$

Can also be formulated as the variance of genetic effects, when the vector of genetic effects is viewed as a vector of fandom variable drawn from the same distribution with mean 0.

Combining everything, we get that $cov(\boldsymbol{y}|\boldsymbol{X\beta}) = \sigma_k^2\boldsymbol{K} + \sigma_e^2\boldsymbol{I}_n$.

Derivation of the two outcomes covariance model.

In the main manuscript, we stated that given two $n \times 1$ vectors $\boldsymbol{y_1}, \boldsymbol{y_2}$, their covariance can be modelled as

$$cov(\boldsymbol{y_1}, \boldsymbol{y_2}) = \sigma_{k,1}\sigma_{k,2}\,\rho_k\,\boldsymbol{K} + \sigma_{e,1}\sigma_{e,2}\rho_e\boldsymbol{I}_n,$$

Where $\boldsymbol{K}$ is the kinship matrix and $\boldsymbol{I}_n = I_{n\times n}$ is the $n \times n$ identity matrix with diagonal values equal to 1 and the rest of the values being equal to zero.

To explain this model, note that $cov(\boldsymbol{y_1}, \boldsymbol{y_2})$ is an $n \times n$ matrix, where the i, j entry provides the covariance $cov(y_{i1}, y_{j2})$ where y_{i1} is outcome 1 of individua i, and y_{j2} is outcome 2 of individual j.

To demonstrate the model, we return to the definition that uses the genetic effects, as in the previous section. Let the vector of genetic effects of outcomes y_1 and y_2 be $\boldsymbol{\alpha}_1$ and $\boldsymbol{\alpha}_2$, respectively.

When $i = j$, i.e. the two outcomes are of the same person, because $k_{ii} = 1$,

$$cov(\boldsymbol{g}_i^T\boldsymbol{\alpha}_1 + e_{i1}, \boldsymbol{g}_i^T\boldsymbol{\alpha}_2 + e_{i2})$$
$$= cov(g_{i1}\alpha_{11} + \cdots + g_{id}\alpha_{d1} + e_{i1}, g_{j1}\alpha_{12} + \cdots + g_{jd}\alpha_{d2} + e_{i2}) =$$
$$= cov(g_{i1}, g_{j1})\alpha_{11}\alpha_{12} + \cdots + cov(g_{id}, g_{jd})\alpha_{21}\alpha_{22} + cov(e_{i1}, e_{i2}) =$$
$$= \sum_{j=1}^{d}\alpha_{j1}\alpha_{j2} + cov(e_{i1}, e_{i2})$$

Treating the vector of variant effect sizes $\boldsymbol{\alpha}_1$ and $\boldsymbol{\alpha}_2$ as vectors of random variables with mean 0, we see that the sum

$$\sum_{j=1}^{d} \alpha_{j1}\alpha_{j2}$$

Expresses the covariance between the elements in the vectors. Covariance of two random variables x_1 and x_2 is defined as: $cov(x_1, x_2) = cor(x_1, x_2) \times \sqrt{var(x_1)var(x_2)}$.
From here follows the model where

$$\sum_{j=1}^{d} \alpha_{j1}\alpha_{j2} = cor(\alpha_1, \alpha_2) \times \sigma_{k1}\sigma_{k2} = \rho_k\sigma_{k1}\sigma_{k2}$$

Where σ_{k1} is defined as the variance of the entries of the $\boldsymbol{\alpha}_1$ vector as before, similarly for α_{k2}, and ρ_k is the genetic correlation between y_1, y_2, formulated as the correlation between the set of genetic effects of the two outcomes.

Similarly,

$$cov(e_{i1}, e_{i2}) = cor(e_{i1}, e_{i2}) = cor(e_1, e_2) \times \sigma_{e1}\sigma_{e2} = \ \rho_e\sigma_{e1}\sigma_{e2}.$$

When $i \neq j$, the derivation is similar. Notable differences are that e_{i1} is independent of e_{j2}, so that $cov(e_{i1}, e_{j2}) = 0$, and that $cov(g_{il}, g_{jl}) = k_{ij}$ rather than 1, for $l = 1, \dots, d$.

$$\begin{aligned} &cov(\boldsymbol{g}_i^T\boldsymbol{\alpha}_1 + \ e_{i1}, \boldsymbol{g}_j^T\boldsymbol{\alpha}_2 + e_{j2}) \\ &\qquad = cov(g_{i1}\alpha_{11} + \dots + \ g_{id}\alpha_{d1} + e_{i1}, g_{j1}\alpha_{12} + \dots + \ g_{jd}\alpha_{d2} + e_{j2}) = \\ &= cov(g_{i1}, g_{j1})\alpha_{11}\alpha_{12} + \dots + \ cov(g_{id}, g_{jd})\alpha_{21}\alpha_{22} + cov(e_{i1}, e_{j2}) = \\ &= k_{ij}\sum_{j=1}^{d}\alpha_{j1}\alpha_{j2} \end{aligned}$$

Therefore:

$$cov(\boldsymbol{g}_i^T\boldsymbol{\alpha}_1 + \ e_{i1}, \boldsymbol{g}_j^T\boldsymbol{\alpha}_2 + e_{j2}) = k_{ij}\rho_k\sigma_{k1}\sigma_{k2}$$

Organizing all the elements in the matrix $cov(\boldsymbol{y_1},\ \boldsymbol{y_2})$, we get that

$$cov(\boldsymbol{y_1},\ \boldsymbol{y_2}) = \sigma_{k,1}\sigma_{k,2}\ \rho_k\ \boldsymbol{K} + \sigma_{e,1}\sigma_{e,2}\rho_e\boldsymbol{I_n}.$$

Supplementary Figures

Figure S1: Estimated coverage probabilities in Settings D and E

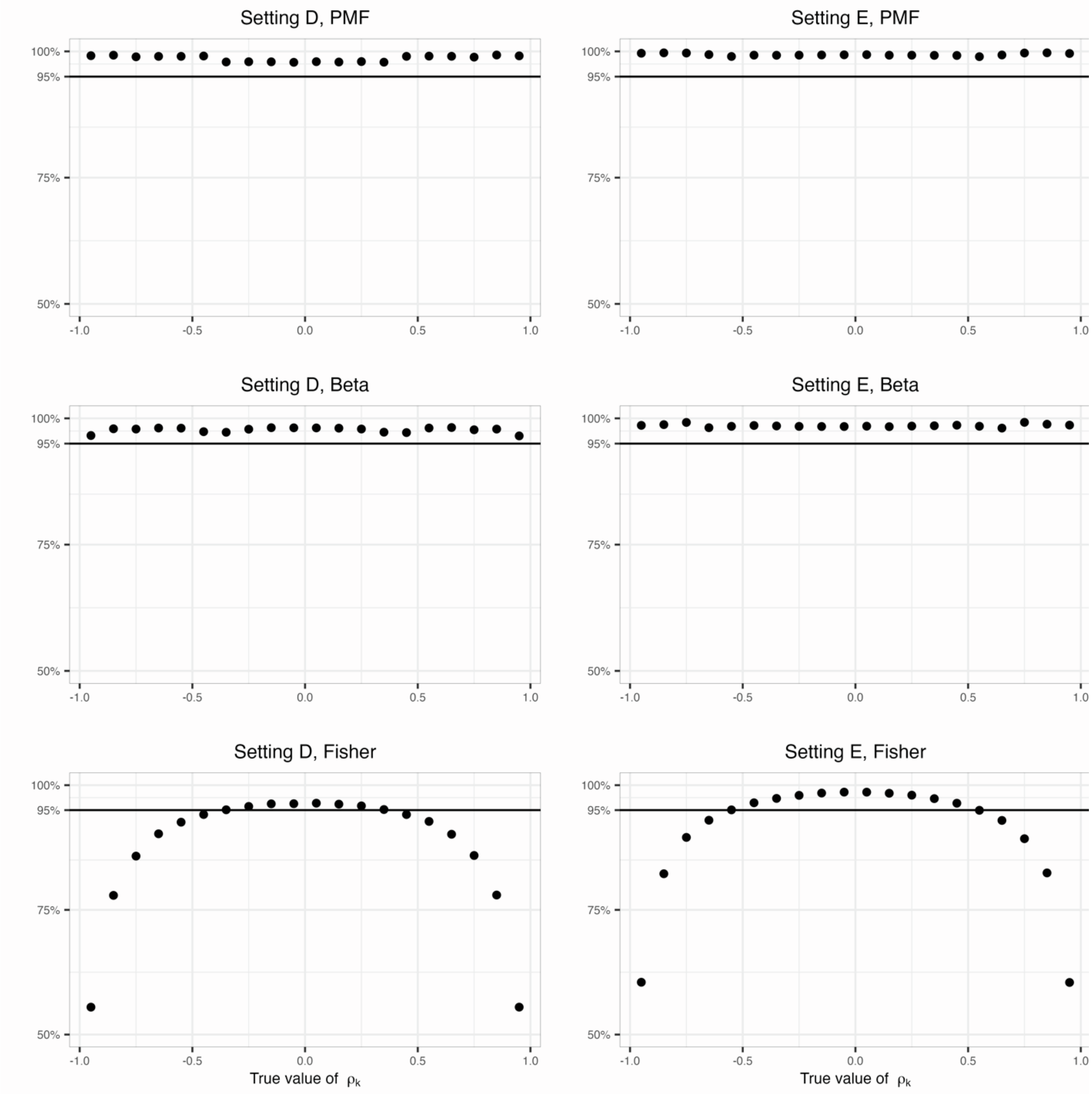

The columns represent different kinship matrix sizes: Setting D denotes the use of the block matrix with three blocks, each in the size all JHS kinship matrix ($3XK_{jhs}$, n=10,254), and Setting E denotes the use of the 10-size kinship matrix ($10XK_{jhs}$, n=34,180). The rows represent three approaches for constructing CIs, including parametric bootstrap PMF, beta approximation for parametric bootstrap PMF, and Fisher's transformation.

Figure S2: Mean CI lengths in Settings D and E

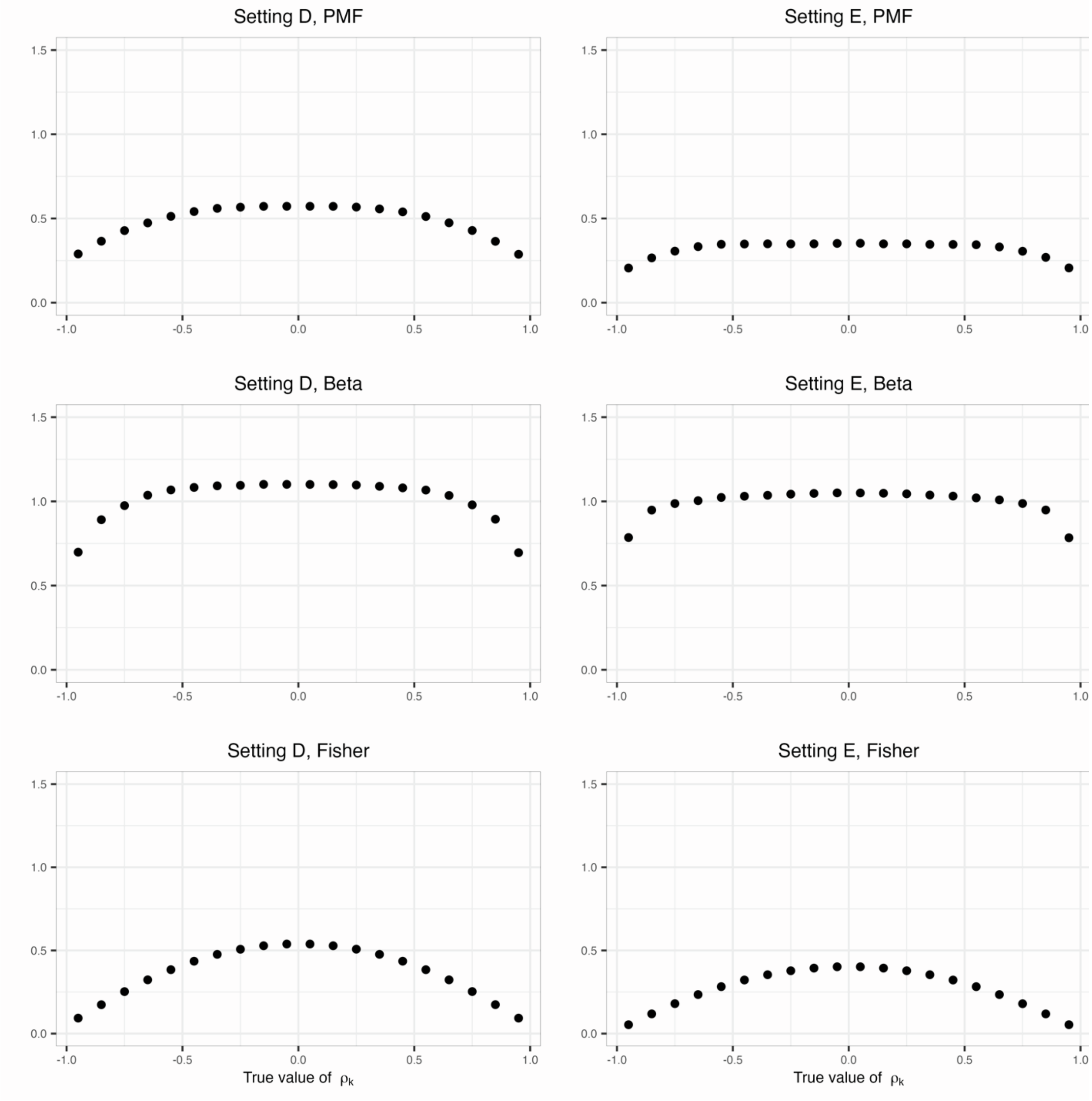

The columns represent different kinship matrix sizes: Setting D denotes the use of the block matrix with three blocks, each in the size all JHS kinship matrix ($3XK_{jhs}$, n=10,254), and Setting E denotes the use of the 10-size kinship matrix ($10XK_{jhs}$, n=34,180). The rows represent three approaches for constructing CIs, including parametric bootstrap PMF, beta approximation for parametric bootstrap PMF, and Fisher's transformation.
