# Supplementary material for: A parametric bootstrap approach for computing confidence intervals for genetic correlations with application to genetically determined protein-protein networks
Source: HGG Adv. 2024 May 8;5(3):100304. doi: 10.1016/j.xhgg.2024.100304 (PMC11140211; doi:10.1016/j.xhgg.2024.100304)
Supplement: Document S3. Article plus supplemental information [file mmc3.pdf]

HGG Advances

ARTICLE

A parametric bootstrap approach for computing confidence intervals for genetic correlations with application to genetically determined protein-protein networks

Yi-Ting Tsai,[1,2,6] Yana Hrytsenko,[1,3,4,6] Michael Elgart,[1,3] Usman A. Tahir,[3,4] Zsu-Zsu Chen,[3,5] James G. Wilson,[3,4] Robert E. Gerszten,[3,4] and Tamar Sofer[1,2,3,4,7,*]

Summary

Genetic correlation refers to the correlation between genetic determinants of a pair of traits. When using individual-level data, it is typically estimated based on a bivariate model specification where the correlation between the two variables is identifiable and can be estimated from a covariance model that incorporates the genetic relationship between individuals, e.g., using a pre-specified kinship matrix. Inference relying on asymptotic normality of the genetic correlation parameter estimates may be inaccurate when the sample size is low, when the genetic correlation is close to the boundary of the parameter space, and when the heritability of at least one of the traits is low. We address this problem by developing a parametric bootstrap procedure to construct confidence intervals for genetic correlation estimates. The procedure simulates paired traits under a range of heritability and genetic correlation parameters, and it uses the population structure encapsulated by the kinship matrix. Heritabilities and genetic correlations are estimated using the close-form, method of moment, Haseman-Elston regression estimators. The proposed parametric bootstrap procedure is especially useful when genetic correlations are computed on pairs of thousands of traits measured on the same exact set of individuals. We demonstrate the parametric bootstrap approach on a proteomics dataset from the Jackson Heart Study.

Introduction

Genetic correlation measures the relationship between a pair of traits through their shared genetic variability.[1] It is a related concept to heritability, which measures the overall genetic contribution to a trait.[2] Specifically, genetic correlation is defined as the correlation between the genetic effects of two traits. It can be estimated using individual-level data or using summary statistics from genome-wide association studies (GWASs).[3] Scientific papers studying the genetic architecture of health and behavioral phenotypes now routinely report genetic correlation estimates between phenotypes, sometimes as a step preceding follow-up analysis, e.g., with polygenic risk scores or Mendelian randomization analyses.[4–7] Genetic correlations are further being studied at the local genomic region level (local genetic correlations), or stratified by genetic annotations, to localize sources of shared genetic underpinnings of phenotypes.[8–12]

Methods for estimating heritability and genetic correlations based on summary statistics from GWASs[3,13,14] became popular in recent years due to their computational tractability and the access to many phenotypes that were interrogated in GWASs by the research community. However, in diverse populations and in small datasets, it is still preferable to estimate heritabilities and genetic correlations using individual-level data, rather than based on GWAS summary statistics.[15] Methods using individual-level data typically rely on an underlying linear mixed model (LMM) formulation, where a genetic relationship matrix is used to model the relationship, or degree of similarity, between the phenotype levels of different individuals.[16,17] When estimating the genetic correlation between two phenotypes, a bivariate normal model is usually used. Common algorithms for estimating heritability and genetic correlation include restricted maximum likelihood (REML)-based normal likelihood models,[18] and method of moment estimators such as the Haseman-Elston approach.[15] Estimation of standard errors (SEs), confidence intervals (CIs), and p values, often relies on asymptotic normal approximation. However, both heritability and genetic correlations have a limited support: heritability is bounded on the [0,1] interval, and genetic correlation on the [−1,1] interval. This means that asymptotic normal approximation may not be appropriate when estimates are close to the boundary of the parameter space, and the problem is more severe with smaller datasets. Previous publications addressed the problem of CI estimation in the context of heritability,[19,20] but, although the distribution of genetic correlations has been studied,[21–23] methods for CI computation in the era of large-scale genomic

[1]Department of Medicine, Brigham and Women's Hospital, Boston, MA, USA; [2]Department of Biostatistics, Harvard T.H. Chan School of Public Health, Boston, MA, USA; [3]Department of Medicine, Harvard Medical School, Boston, MA, USA; [4]CardioVascular Institute (CVI), Beth Israel Deaconess Medical Center, Boston, MA, USA; [5]Department of Internal Medicine, Division of Endocrinology, Diabetes, and Metabolism, Beth Israel Deaconess Medical Center, Boston, MA, USA
[6]These authors equally contributed
[7]Lead contact
*Correspondence: tsofer@bidmc.harvard.edu
https://doi.org/10.1016/j.xhgg.2024.100304.
© 2024 The Author(s). This is an open access article under the CC BY license (http://creativecommons.org/licenses/by/4.0/).

studies have not been as developed. Notably, we previously proposed a Fisher's transformation-based approach and a blocked bootstrap, relying on resampling from the data, by blocks of related individuals.[15] The blocked bootstrap worked better than the Fisher's transformation approach, but was computationally more intensive; we, therefore, only allowed for a small number of resamples, limiting the potential coverage of the CIs as well as application at scale (i.e., for millions of traits). Here, we build on a prior work by Schweiger et al.[19] in the context of heritability. We expand their parametric bootstrap test-inversion method that eliminates the dependency on asymptotic approximation.

In this paper, we develop a parametric bootstrap approach to construct CIs for genetic correlations to better model the unknown distribution of genetic correlations. The procedure requires simulating pairs of phenotypes using the existing correlation structure between individuals in a given dataset, based on sets of values of heritabilities and genetic correlation between the phenotypes. The results from the simulation study are used to construct CIs for the genetic correlation parameter based on triplets of estimated heritabilities and the genetic correlation of a pair of phenotypes, using the conditional empirical probability mass function (PMF) of the genetic correlation parameter. We demonstrate and compare, through simulations, the performance of two variations of the parametric bootstrap procedure and further compare them with construction of CIs based on the Fisher's transformation of the estimated genetic correlation, and estimated SEs of the correlation parameter from asymptotic normal assumption on REML estimates. Despite being a resampling method, typically requiring many computations and thus computationally costly, our approach is very useful when estimating genetic correlations between thousands of traits measured on the same dataset, because the simulation study used to construct PMFs is performed once and may be used many times. Thus, we demonstrate the application of the parametric bootstrap approach to study genetic correlations between a high-dimensional set of proteins and to develop protein-protein networks based on the genetic correlations estimated in the Jackson Heart Study (JHS) dataset.

Subjects, material, and methods

LMM formulation

Let $\boldsymbol{y}$ be an $n\times1$ phenotype outcome vector and $\boldsymbol{X}$ be an $n\times p$ matrix containing values of p covariates measured on n participants. Let $\boldsymbol{e}$ be an $n\times1$ vector of residuals, or errors, which we assume are potentially correlated across participants due to shared genetic effects. Suppose that the $n\times n$ matrix $\boldsymbol{K}$ models the genetic relationship between individuals, such that its i,j entry $k_{i,j}$ is, for example, (twice) the kinship coefficient between individual i and j, and $k_{j,i} = k_{i,j}$ (i.e., this is a symmetric matrix). Note that genetic relationship could be estimated by various quantities,[24] without loss of generality; we here assume that we use a kinship matrix using identity by descent estimates. Following standard LMM formulation of heritability, we model the outcome as

$$\boldsymbol{y} = \boldsymbol{X\beta} + \boldsymbol{G\alpha} + \boldsymbol{e},$$

where β are the regression coefficients of the covariates, here treated as nuisance parameters, $\boldsymbol{G}$ is a matrix of genetic variants, and $\boldsymbol{\alpha}$ is the vector with variant effects on the outcome. In the Supplementary Note, we demonstrate how genetic effects contributing to the phenotypic variance, so that, given covariate effects, the total variance is decomposed to genetic variance and remaining residual variance. Let σ_k^2 be the genetic variance component, and σ_e^2 be the residual variance component, so that

$$\boldsymbol{y} \sim N\left(\boldsymbol{X\beta}, \sigma_k^2\boldsymbol{K} + \sigma_e^2\boldsymbol{I}_n\right).$$

The narrow-sense heritability, defined as the proportion of total variance explained by additive genetic factors is:

$$h^2 = \frac{\sigma_k^2}{\sigma_k^2 + \sigma_e^2}.$$

Here, we assume, without loss of generality, that the total variance $\sigma_T^2 = \sigma_k^2 + \sigma_e^2 = 1$. This is a reasonable assumption because the heritability does not depend on the value of the total variance and the outcome y can be rescaled to have total variance of 1 and heritability estimates are not affected (as both σ_k^2 and $\sigma_k^2 + \sigma_e^2$ are divided by the same value). Therefore, we have $\sigma_k^2 = h^2$, meaning that the genetic variance is equal to the heritability. Under this assumption, the variance of the phenotype can be written as

$$var(\boldsymbol{y}) = \sigma_k^2\boldsymbol{K} + (1 - \sigma_k^2)\boldsymbol{I_n}.$$

Given two $n\times1$ vectors $\boldsymbol{y_1}, \boldsymbol{y_2}$, their covariance can be modeled as

$$cov(\boldsymbol{y_1}, \boldsymbol{y_2}) = \sigma_{k,1}\sigma_{k,2}\,\rho_k\,\boldsymbol{K} + \sigma_{e,1}\sigma_{e,2}\rho_e\boldsymbol{I_n},$$

where $\sigma_{k,i}^2$ is the genetic variance for phenotype $i\in\{1,2\}$, $\sigma_{e,i}^2$ is the residual variance for phenotype i, ρ_k is the genetic correlation between the two phenotypes, and ρ_e is the residual correlation between the two phenotypes.[15] More details are provided in the Supplementary Note. Alternatively:

$$cov(\boldsymbol{y_1}, \boldsymbol{y_2}) = \sigma_{k,1}\sigma_{k,2}\,\rho_k\,\boldsymbol{K} + \sqrt{1 - \sigma_{k,1}^2}\sqrt{1 - \sigma_{k,2}^2}\,\rho_e\,\boldsymbol{I_n}. \quad \text{(Equation 1)}$$

If we further plug in $\sigma_k^2 = h^2$, then, for a single and for two phenotypes, we can write the variance model as:

$$var(\boldsymbol{y}) = h^2\boldsymbol{K} + \left(1 - h^2\right)\boldsymbol{I_n} \quad \text{(Equation 2)}$$

$$cov(\boldsymbol{y_1}, \boldsymbol{y_2}) = h_1h_2\,\rho_k\,\boldsymbol{K} + \sqrt{1 - h_1^2}\sqrt{1 - h_2^2}\,\rho_e\,\boldsymbol{I_n}, \quad \text{(Equation 3)}$$

which is the form that we will use to simulate outcomes in the following parametric bootstrap section.

Parametric bootstrap

We use a parametric bootstrap approach to compute CIs. In brief, we simulate data for each set of potential values of heritabilities $\tilde{h}_1^2, \tilde{h}_2^2$ and genetic and residual correlation $\tilde{\rho}_k, \tilde{\rho}_e$ between two phenotypes based on the existing genetic relationship between individuals in the dataset of interest. Next, we compute CIs by inferring ranges of potential values of ρ_k (integrated over potential values of h_1^2, h_2^2, as the true values are not known) that resulted in realized (estimated) values $\widehat{h}_1^2, \widehat{h}_2^2, \widehat{\rho}_k$. In practice, to limit computational burden, we fix $\tilde{\rho}_e = 0$ (and we assess the use of $\tilde{\rho}_e = 0.2, 0.4$ also as fixed values in simulations). Figure 1 visualizes the parametric bootstrap approach for computing CIs: the first step is the parametric bootstrap itself, where simulations are performed based on set heritabilities and genetic correlation values and results from all simulations are collected and stored. The second step is the computation of CIs for a given triplet of estimated heritabilities and genetic correlation values, based on a PMF computed from the stored parametric bootstrap results.

Step 1: Random sampling of genetically correlated outcomes

For every given combination of the potential heritability of phenotype 1 ($\tilde{h}_1^2$), potential heritability of phenotype 2 ($\tilde{h}_2^2$), and potential genetic correlation ($\tilde{\rho}_k$), we draw N (e.g., 10,000) pairs of phenotype vectors ($\boldsymbol{y_1}, \boldsymbol{y_2}$) from the multivariate normal distribution

$$\begin{pmatrix} \boldsymbol{y_1} \\ \boldsymbol{y_2} \end{pmatrix} \sim N\left[\mathbf{0}_{2n \times 1}, \begin{pmatrix} var(\boldsymbol{y_1}) & cov(\boldsymbol{y_1}, \boldsymbol{y_2}) \\ cov(\boldsymbol{y_1}, \boldsymbol{y_2}) & var(\boldsymbol{y_2}) \end{pmatrix}\right],$$

where

$$\begin{cases} var(\boldsymbol{y_1}) = \tilde{h}_1^2 \boldsymbol{K} + \left(1 - \tilde{h}_1^2\right) \boldsymbol{I}_n \\ var(\boldsymbol{y_2}) = \tilde{h}_2^2 \boldsymbol{K} + \left(1 - \tilde{h}_2^2\right) \boldsymbol{I}_n \\ cov(\boldsymbol{y_1}, \boldsymbol{y_2}) = \tilde{h}_1 \tilde{h}_2 \, \tilde{\rho}_k \, \boldsymbol{K} + \sqrt{1 - \tilde{h}_1^2} \sqrt{1 - \tilde{h}_2^2} \, \tilde{\rho}_e \, \boldsymbol{I}_n \end{cases}$$

where $\tilde{h}_1^2, \tilde{h}_2^2 \in [0, 1]$ and $\tilde{\rho}_k \in [-1, 1]$. We note here that ρ_e may take potential value in the interval $[-1, 1]$, but we choose just one value, as mentioned earlier. We used 10 settings for $\tilde{h}_1^2$, 10 settings for $\tilde{h}_2^2$, and 20 settings for $\tilde{\rho}_k$ as follows:

$$\tilde{h}_1 = \{0.05, 0.15, 0.25, \ldots, 0.85, 0.95\}$$

$$\tilde{h}_2 = \{0.05, 0.15, 0.25, \ldots, 0.85, 0.95\}$$

$$\tilde{\rho}_k = \{-0.95, -0.85, \ldots, -0.05, 0.05, 0.15, \ldots, 0.85, 0.95\}.$$

Under this setup, there are 2,000 distinct combinations of triplets ($\tilde{h}_1^2$, $\tilde{h}_2^2$, $\tilde{\rho}_k$) in total. We note that while developing this procedure we compared using finer grids of values, with sequences with differences of 0.01 between each two consecutive values, but the results remained essentially the same while the computational burden was substantially higher. Because the grid size determines the accuracy level of potential CI coverage, we later offer a solution to increase coverage without simulating a finer grid of values.

Step 2: Genetic correlation and heritability estimation

Next, based on each sampled pair of phenotype vectors ($\boldsymbol{y_1}$, $\boldsymbol{y_2}$), we estimate $\widehat{h}_1^2$, $\widehat{h}_2^2$, $\widehat{\rho}_k$. While the procedure is in principle naive to the specific formula used, we are using the closed-form Hasemen-Elson formulas we previously derived[15,20]:

$$\widehat{h}_l^2 = \frac{\boldsymbol{y_l^T W y_l}}{tr(\boldsymbol{WW})}, l \in \{1, 2\} \qquad \text{(Equation 4)}$$

$$\widehat{\rho}_k = \frac{\boldsymbol{y_1^T W y_2}}{\sqrt{\boldsymbol{y_1^T W y_1}}\sqrt{\boldsymbol{y_2^T W y_2}}}, \qquad \text{(Equation 5)}$$

where $\boldsymbol{W}$ is either the kinship matrix with all diagonal values set to zero or a weighted sum of the kinship matrix $\boldsymbol{K}$ and the matrix modeling the random error (here, an identity matrix) with weights related to the relationship between the kinship matrix and the identity matrix. See Elgart et al.[15] for more details, including the potential use of multiple matrices modeling correlations between individuals. In practice, it is appropriate to use the kinship matrix with the diagonal value set to zero when only the kinship matrix is used to model the relationship between individuals. Using these formulas rather than likelihood-based procedures is computationally quicker, as no iterations are required.

Step 3: PMF estimation for $Pr(\rho_k | \widehat{h}_1^2, \widehat{h}_2^2, \widehat{\rho}_k^2)$

We now derive the expression for the conditional probability of ρ_k given the estimated parameters. Because the support of h_1^2, h_2^2, ρ_k is continuous, where $h_1, h_2 \in [0, 1]$ and $\rho_k \in [-1, 1]$, while the results from simulations are discrete values, we divide these ranges into bins, e.g., of size 0.1, i.e., forcing them into a discrete distribution:

$$\rho_k \in \{\mathcal{A}_1^\rho = [-1, -0.9), \mathcal{A}_2^\rho = [-0.9, -0.8), \ldots, \mathcal{A}_{19}^\rho = [0.8, 0.9), \mathcal{A}_{20}^\rho = [0.9, 1]\}.$$

$$h_1, h_2 \in \{\mathcal{A}_1^h = [0, 0.1), \mathcal{A}_2^h = [0.1, 0.2), \ldots, \mathcal{A}_9^h = [0.8, 0.9), \mathcal{A}_{10}^h = [0.9, 1]\}.$$

When estimating CIs for genetic correlations, we are given the estimates $\widehat{h}_1^2, \widehat{h}_2^2$, $\widehat{\rho}_k$ and we want to identify a region $\mathcal{A}$ such that $\Pr(\tilde{\rho}_k \in \mathcal{A} | \widehat{h}_1 \in \mathcal{A}_i^h, \widehat{h}_2 \in \mathcal{A}_j^h, \widehat{\rho}_k \in \mathcal{A}_k^\rho) = 1 - \alpha$ (e.g., $1 - \alpha = 95\%$). Therefore, we want to estimate the probabilities $\Pr(\tilde{\rho}_k \in \mathcal{A}_i^\rho | \widehat{h}_1^2 \in \mathcal{A}_j^h, \widehat{h}_2^2 \in \mathcal{A}_k^h, \widehat{\rho}_k \in \mathcal{A}_l^\rho)$ for $i = 1, \ldots, 20$ to create an empirical PMF and use it to generate CIs, which can be derived using Bayes theorem. The derivation below uses the probabilities $\Pr(\widehat{h}_1^2 \in \mathcal{A}_i^h | \tilde{h}_1^2 \in \mathcal{A}_j^h)$, $\Pr(\widehat{h}_2^2 \in \mathcal{A}_i^h | \tilde{h}_2^2 \in \mathcal{A}_j^h)$, $\Pr(\widehat{\rho}_k \in \mathcal{A}_i^\rho | \tilde{h}_1^2 \in \mathcal{A}_j^h, \tilde{h}_2^2 \in \mathcal{A}_k^h, \tilde{\rho}_k \in \mathcal{A}_l^\rho)$, which are the probabilities of $\widehat{h}_1, \widehat{h}_2, \widehat{\rho}_k$ being in given regions conditional on the fixed values of $\tilde{h}_1^2, \tilde{h}_2^2, \tilde{\rho}_k$ (note that the probabilities of the

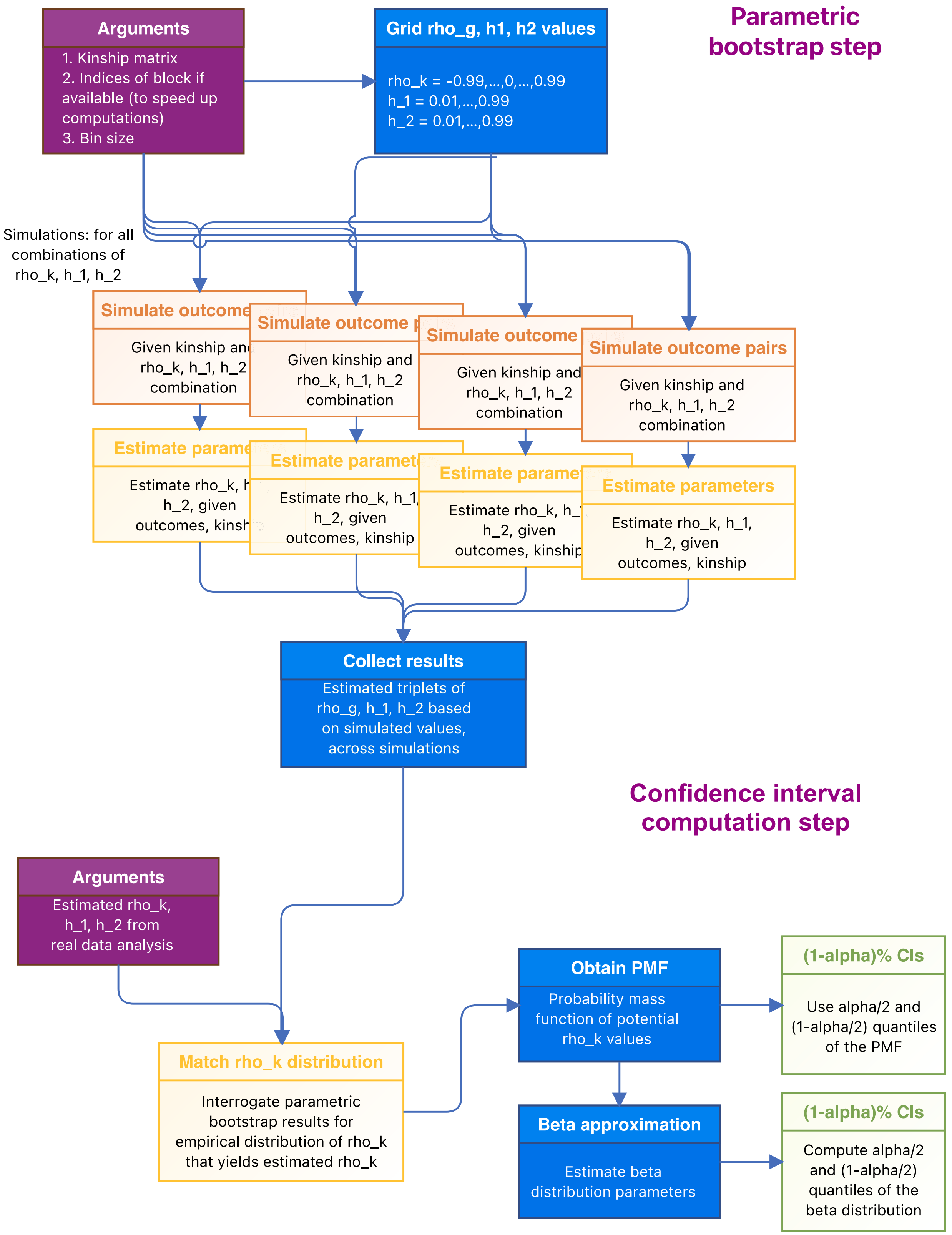

Figure 1. Parametric bootstrap approach for generating CIs for genetic correlations
The figure visualizes the parametric bootstrap approach for computing CIs for genetic correlations. The parametric bootstrap itself simulates data from the correlation structure (modeled via the genetic relationship, or kinship, matrix), applies the analytic procedure for computing heritabilities and genetic correlations, and stores the results. The CI computation step relies on the results from the parametric bootstrap. It estimates CIs based on parameter values that may result in the observed associations.

estimated heritabilities do not depend on the heritabilities of other traits on or the genetic correlation between them). Moving forward, we drop the notations showing that values refer to bins (regions) for brevity, with the understanding that all probabilities refer to parameters being in bins. Therefore, we will denote $\Pr(\tilde{\rho}_k | \hat{\rho}_k, \hat{h}_1^2, \hat{h}_2^2)$ instead of $\Pr(\tilde{\rho}_k \in \mathcal{A}_i^\rho | \hat{\rho}_k \in \mathcal{A}_j^\rho, \hat{h}_1^2 \in \mathcal{A}_k^h, \hat{h}_2^2 \in \mathcal{A}_l^h)$, etc.

We first note that $\Pr(\tilde{\rho}_k | \hat{\rho}_k, \hat{h}_1^2, \hat{h}_2^2) = \frac{\Pr(\tilde{\rho}_k, \hat{\rho}_k, \hat{h}_1^2, \hat{h}_2^2)}{\Pr(\hat{\rho}_k, \hat{h}_1^2, \hat{h}_2^2)}$, and therefore, we need to estimate $\Pr(\tilde{\rho}_k, \hat{\rho}_k, \hat{h}_1^2, \hat{h}_2^2)$ and $\Pr(\hat{\rho}_k, \hat{h}_1^2, \hat{h}_2^2)$.

Estimating $\Pr(\hat{\rho}_k, \hat{h}_1^2, \hat{h}_2^2)$

We estimate $\Pr(\hat{\rho}_k, \hat{h}_1^2, \hat{h}_2^2)$ based on the following:

$$\Pr\left(\hat{\rho}_k, \hat{h}_1^2, \hat{h}_2^2\right) = \sum_{\tilde{\rho}_k}\sum_{\tilde{h}_1}\sum_{\tilde{h}_2}\Pr\left(\hat{\rho}_k, \hat{h}_1^2, \hat{h}_2^2, \tilde{\rho}_k, \tilde{h}_1^2, \tilde{h}_2^2\right) =$$

$$= \sum_{\tilde{\rho}_k}\sum_{\tilde{h}_1}\sum_{\tilde{h}_2}\Pr\left(\hat{\rho}_k, \hat{h}_1^2, \hat{h}_2^2 | \tilde{\rho}_k, \tilde{h}_1^2, \tilde{h}_2^2\right)\Pr\left(\tilde{\rho}_k, \tilde{h}_1^2, \tilde{h}_2^2\right) =$$

$$= \frac{1}{n_\rho n_h^2}\sum_{\tilde{\rho}_k}\sum_{\tilde{h}_1}\sum_{\tilde{h}_2}\Pr\left(\hat{\rho}_k, \hat{h}_1^2, \hat{h}_2^2 | \tilde{\rho}_k, \tilde{h}_1^2, \tilde{h}_2^2\right),$$

where, for bins of length 0.1, $n_\rho = 20$, $n_h = 10$.

Estimating $\Pr(\tilde{\rho}_k, \hat{\rho}_k, \hat{h}_1^2, \hat{h}_2^2)$

We estimate $\Pr(\tilde{\rho}_k, \hat{\rho}_k, \hat{h}_1^2, \hat{h}_2^2)$ based on the following:

$$\Pr\left(\tilde{\rho}_k, \hat{\rho}_k, \hat{h}_1^2, \hat{h}_2^2\right) = \Pr\left(\hat{\rho}_k, \hat{h}_1^2, \hat{h}_2^2 | \tilde{\rho}_k\right)\Pr(\tilde{\rho}_k) = \frac{1}{n_\rho}\Pr\left(\hat{\rho}_k, \hat{h}_1^2, \hat{h}_2^2 | \tilde{\rho}_k\right) =$$

$$= \frac{1}{n_\rho}\sum_{\tilde{h}_1}\sum_{\tilde{h}_2}\Pr\left(\hat{\rho}_k, \hat{h}_1^2, \hat{h}_2^2, \tilde{h}_1^2, \tilde{h}_2^2 | \tilde{\rho}_k\right) =$$

$$= \frac{1}{n_\rho}\sum_{\tilde{h}_1}\sum_{\tilde{h}_2}\Pr\left(\hat{\rho}_k, \hat{h}_1^2, \hat{h}_2^2 | \tilde{\rho}_k, \tilde{h}_1^2, \tilde{h}_2^2\right)\Pr\left(\tilde{h}_1^2, \tilde{h}_2^2 | \tilde{\rho}_k\right) =$$

$$= \frac{1}{n_\rho}\sum_{\tilde{h}_1}\sum_{\tilde{h}_2}\Pr\left(\hat{\rho}_k, \hat{h}_1^2, \hat{h}_2^2 | \tilde{\rho}_k, \tilde{h}_1^2, \tilde{h}_2^2\right)\Pr\left(\tilde{h}_1^2\right)\Pr\left(\tilde{h}_2^2\right) =$$

$$= \frac{1}{n_\rho n_h^2}\sum_{\tilde{h}_1}\sum_{\tilde{h}_2}\Pr\left(\hat{\rho}_k, \hat{h}_1^2, \hat{h}_2^2 | \tilde{\rho}_k, \tilde{h}_1^2, \tilde{h}_2^2\right)$$

Putting these together:

$$\Pr\left(\tilde{\rho}_k | \hat{\rho}_k, \hat{h}_1^2, \hat{h}_2^2\right) = \frac{\Pr\left(\tilde{\rho}_k, \hat{\rho}_k, \hat{h}_1^2, \hat{h}_2^2\right)}{\Pr\left(\hat{\rho}_k, \hat{h}_1^2, \hat{h}_2^2\right)} = \frac{\sum_{\tilde{h}_1}\sum_{\tilde{h}_2}\Pr\left(\hat{\rho}_k, \hat{h}_1^2, \hat{h}_2^2 | \tilde{\rho}_k, \tilde{h}_1^2, \tilde{h}_2^2\right)}{\sum_{\tilde{\rho}}\sum_{\tilde{h}_1}\sum_{\tilde{h}_2}\Pr\left(\hat{\rho}_k, \hat{h}_1^2, \hat{h}_2^2 | \tilde{\rho}_k, \tilde{h}_1^2, \tilde{h}_2^2\right)}$$

$\Pr(\rho_k | \hat{\rho}_k, \hat{h}_1^2, \hat{h}_2^2)$ (computed for each pre-defined region) is then the empirical PMF of ρ_k obtained by parametric bootstrap.

Computing CIs from the PMF

After obtaining the empirical PMF from parametric bootstrap, we can now derive the CIs for any given genetic correlation estimate $\hat{\rho}_k$ with a coverage probability of $1 - \alpha$ (e.g., 95%). Because the parameters are bounded, constructed CIs may be asymmetric in both the distance between the estimated $\hat{\rho}_k$ to the low and high values of the CI, and in the cumulative probability between provided by the two "sides" (around $\hat{\rho}_k$) of the CI. We address this by considering the following three cases depending on the cumulative probability:

$$cp_l = \sum_{\rho_k \le \hat{\rho}_k}\Pr\left(\rho_k | \hat{\rho}_k, \hat{h}_1^2, \hat{h}_2^2\right).$$

Here, cp_l denotes cumulative probability of potential ρ_k values lower or equal to the estimated $\hat{\rho}_k$. Denote the low and the high values of the CI for $\hat{\rho}_k$ by $\hat{\rho}_L$ and $\hat{\rho}_H$. Then a 1-α CI news to include all potential values $\tilde{\rho}_k$ such that:

$$\sum_{\hat{\rho}_L \le \tilde{\rho}_k \le \hat{\rho}_H}\Pr\left(\tilde{\rho}_k | \hat{\rho}_k, \hat{h}_1^2, \hat{h}_2^2\right). \qquad \text{(Equation 6)}$$

Case 1: If $cp_l < \frac{1-\alpha}{2}$

Here, $\hat{\rho}_L$ corresponds with the first potential value $\tilde{\rho}_k$ (i.e., a point in the first considered bin, where bins are considered by order $\mathcal{A}_1^\rho, \mathcal{A}_2^\rho \ldots$), where $\Pr(\tilde{\rho}_k | \hat{\rho}_k, \hat{h}_1^2, \hat{h}_2^2) > 0$. $\hat{\rho}_H$ corresponds with the smallest potential value $\tilde{\rho}_k$ satisfying equation.[6]

Case 2: If $1 - cp_l < \frac{1-\alpha}{2}$

In this case, we first identify $\hat{\rho}_H$ as the highest $\tilde{\rho}_k$ (i.e., a point in first considered bin, where bins are considered by order $\mathcal{A}_{n_h}^\rho, \mathcal{A}_{n_h-1}^\rho \ldots$) with $\Pr(\tilde{\rho}_k | \hat{\rho}_k, \hat{h}_1^2, \hat{h}_2^2) > 0$. $\hat{\rho}_L$ corresponds to the highest potential value $\tilde{\rho}_k$ satisfying equation.[6]

Case 3: Both $cp_l > \frac{1-\alpha}{2}$ and $1 - cp_l > \frac{1-\alpha}{2}$

Here we require $\hat{\rho}_L$ to be the largest value and $\hat{\rho}_H$ to be the lowest such that $\sum_{\hat{\rho}_L \le \tilde{\rho}_k}\Pr(\tilde{\rho}_k | \hat{\rho}_k, \hat{h}_1^2, \hat{h}_2^2)$, $\sum_{\tilde{\rho}_k \ge \hat{\rho}_H}\Pr(\tilde{\rho}_k | \hat{\rho}_k, \hat{h}_1^2, \hat{h}_2^2) \ge \frac{1-\alpha}{2}$.

When the upper bound or lower bound of CIs obtained from the above procedure lies somewhere inside the bins defined by the grid of considered values, which is often the case, we use linear interpolation to get a position for upper and lower bound as point within the bins.

Empirical beta approximation to the PMF for CI estimation

Because the PMF is discrete, it limits the potential coverage of constructed CIs and the potential computation of accurate p values. Thus, we study a continuous beta approximation to the empirical PMF from parametric bootstrap. The goal is to use this approach when CIs with high coverage are sought, and when low p values need to be inferred

(see below). Since the range of genetic correlation is [−1, 1], and the range of beta distribution is [0, 1], we first map the [−1, 1] range of genetic correlations to the [0, 1] range of beta distribution through a location-scale transformation. After finding the 100*(1 − α)% CIs of $\widehat{\rho}_k$ on the beta scale using a similar approach to that reported based on the discreate PMF, we apply the inverse location-scale transformation from [0, 1] to [−1, 1] to retrieve the CIs of genetic correlations.

p Value estimation

We evaluate the use of the CI inversion method to obtain p values for hypothesis testing. Here, our null hypothesis H_0 is $\rho_k = 0$, and our alternative hypothesis H_1 is $\rho_k \neq 0$. Given any realization $(\widehat{h}_1, \widehat{h}_2, \widehat{\rho})$, we can estimate the CIs for ρ_k using the parametric bootstrap procedure, focusing on the continuous beta approximation to the empirical PMF because smaller accurate p values can be obtained if the underlying distribution is continuous. To determine the p values of genetic correlation estimates, we use the CI inversion methods. Suppose that we construct a 100 ×(1 − α)% CI. Then, we can determine that the p value is smaller than α if the constructed CI does not cover 0. For computational efficiency, we implemented a method that constructs CIs using a binary search approach to the α value, stopping when a pre-defined sensitivity level is reached. In more detail, the binary search relies on the idea of choosing and α value, computing a CI, and checking whether 0 is within the interval, i.e., it relies on a function that gets an α value and return "yes" or "no" (1 or 0) if the CI does or does not cover zero. The search is to find the smallest α value where the CI does not cover zero.

The JHS

The JHS is a longitudinal study following 5,306 individuals of African American background from Jackson, Mississippi.[25,26] The study population includes 2,050 related and unrelated JHS participants who had whole genome sequencing (WGS) through the Trans-Omics for Precision Medicine (TOPMed) program, proteomics data, and available body mass index (BMI). The TOPMed Data Coordinating Center used TOPMed WGS data from the TOPMed freeze 8 release and computed kinship matrix, tabulating the genetic relationship between TOPMed participants. We subsetted this matrix into JHS participants. Concentration levels of 1,317 proteins in plasma were measured using the SomaScan platform.[27] The JHS study followed procedures in accordance with the ethical standards of the responsible committee. It was approved by Jackson State University, Tougaloo College, and the University of Mississippi Medical Center Institutional Review Boards, and all participants provided written informed consent.

We excluded five proteins with more than 80% missing values. The remaining dataset had no missing protein measurements. Protein measurements were adjusted for batch effect by rank-normalizing each protein separately in each batch and then aggregating the data across batches. Next, the protein measurements were regressed over (a) only the intercept (i.e., the measurements were centered, and the analysis not adjusted for covariates), and (b) over age, sex, and BMI. The residuals from each of these regressions were extracted and were used for estimating heritabilities and genetic correlations between all protein pairs using Haseman-Elston estimators provided in Equations 4 and 5. In addition, we compared the estimates of genetic correlations with estimated Pearson correlations calculated using *stats* R package (version 3.6.2).

Simulation studies

We used the kinship matrix from the JHS data to perform simulations. It is important to clarify that there are two separate types of simulations: first, for a given dataset (including a synthetic dataset), there are simulations that are done as part of the parametric bootstrap step (as in the first, top, step in Figure 1). Second, we perform simulation studies to evaluate the statistical methodology. For these, we generate data and use it for the second step in Figure 1: we use the parameters estimated in each simulation repetition and the parametric bootstrap to compute CIs. Thus, we used the kinship matrix from the JHS data to both perform parametric bootstrap (which includes simulations) and next to generate data that next used the parametric bootstrap results.

To study methods' performance in larger sample sizes, we also created synthetic datasets mimicking the JHS in which we used block matrices, with blocks being the original JHS kinship matrix using n = 2,050 individuals (setting A). We used two and three times the original sample size to form block diagonal kinship matrices with n = 4,100 and n = 6,150 (settings B and C). Thus, we used these kinship matrices to (a) perform simulations for the parametric bootstrap procedure, where in the primary simulations we fix $\tilde{\rho}_e = 0.2$ as a conservative potential high value of ρ_e. We also performed simulations comparing the choice of $\tilde{\rho}_e \in \{0, 0.2, 0.4\}$. Next, (b) we generate new simulated data that used the results of the parametric bootstrap simulations (a) to construct CIs. We performed 10,000 simulations for each combination of potential (h_1^2, h_2^2, ρ_k), with $h_1^2, h_2^2 \in \{0.05, 0.15, \ldots, 0.95\}$ and $\rho_k \in \{-0.95, -0.85, \ldots, -0.05, 0.05, \ldots, 0.095\}$. We constructed CIs for the estimated $\widehat{\rho}_k$ in each simulation.

To further assess computational performance in larger sample sizes, we also performed limited analyses where we used the full JHS dataset available in TOPMed freeze 8 (n = 3,418), and created a block diagonal kinship matrix with 3 and 10 times this matrix, with n = 10,254 and n = 34,180 (settings D and E). In these simulations, we had $\tilde{\rho}_e = 0$, and assessed CI computation performance using a small number of 1,000 simulations for each combination of potential (h_1^2, h_2^2, ρ_k).

Comparison: Four approaches for constructing CIs

We estimated the coverage and the width of the CIs constructed using the two proposed approaches and two

existing approaches: (a) percentiles of the empirical PMF constructed using the parametric bootstrap approach (proposed); (b) beta approximation to the empirical PMF (proposed); and the existing methods: (c) Fisher's transformation; and (d) normal approximation to the distribution of the estimated genetic correlation implemented in the genome-wide complex trait analysis (GCTA) package.[28]

The Fisher's transformation method assumes that genetic correlations follow the same distributions as Pearson correlations.[29] More specifically, they are normally distributed after Fisher's transformation. For genetic correlation ρ_k,

$$z = \frac{1}{2}\ln\left(\frac{1+\rho_k}{1-\rho_k}\right) \sim \mathrm{N}(\mu, \sigma), where$$

$$\mu = \frac{1}{2}\ln\left(\frac{1+\rho_k}{1-\rho_k}\right), \sigma = \frac{1}{\sqrt{N_{eff}-3}},$$

With N_{eff} being the "effective sample size," previously proposed to be $trace(\boldsymbol{K}^{-}\boldsymbol{K}^{-})$, with $\boldsymbol{K}^{-}$ being the kinship matrix with diagonal values set to zero.[15] We can then construct the CIs of z by the standard approach, assuming asymptotic normal distributions. For example, the 95% CI of z would be $[\mu - 1.96\sigma, \ \mu + 1.96\sigma]$. After finding the $100*(1-\alpha)\%$ CIs of $\hat{\rho}_k$ on the Fisher's transformed (z) scale, we apply the inverse Fisher's transform to retrieve the CIs of the genetic correlation ρ_k.

To compute CIs based on existing approach that rely on a normal approximation, we estimate both the genetic correlation and its SE using the bivariate REML procedure implemented in the GCTA package. We apply the $[\hat{\rho} - 1.96\,SE, \ \hat{\rho} + 1.96\,SE]$ formula to construct 95% CIs. Due to the high computational resources required by GCTA, we focus only on the four scenarios when true ρ_k equals {0.05, 0.15, 0.45, 0.95} with the original-size kinship matrix.

Performance evaluation metrics

We used coverage probabilities and CI widths as the metrics to evaluate and compare the performance of the four approaches for CI construction. In primary results, for a given true value of genetic correlation ρ_k we calculated both the coverage probability and the average width of 95% CIs using the constructed CIs for the estimated ρ_k over all the 100 true heritability scenarios (10 for h_1^2 and 10 for h_2^2). Ideally, the coverage probabilities would be at or above 95% across different ρ_k, and also having small CI widths. The coverage probability was estimated as the proportion of simulations in which the true ρ_k was contained in its CI.

Type 1 error when testing genetic correlations

For each combination of potential heritability values ($\tilde{h}_1^2$, $\tilde{h}_2^2$), we simulated 10,000 pairs of phenotype vectors ($\boldsymbol{y_1}$, $\boldsymbol{y_2}$) under the null, i.e., $\rho = 0$, estimated their genetic correlations, and calculated their p values as described above based on the beta approximation to the PMF. After obtaining the p values for all the 10,000 simulated data, we estimated the type 1 error rate, also called the size of the test, by checking the percentage of these simulation rejecting the null given an α value.

Computational optimizations and assessments

The computational burden for performing parametric bootstrap increases with the size of the kinship matrix. We implemented a few approaches to optimize computations, all of which rely on the realistic assumption that the kinship matrix can be ordered by blocks, where individuals in different blocks are unrelated to each other. In other words, the kinship coefficient describing relatedness between individuals in different blocks is set to zero, to create a block matrix. While this may lead to some loss of information (as genetic correlation estimation using individual-level data relies on genetic relatedness between individuals), this approach is standard in the analysis of large genetic datasets,[30] because it balances the low contribution of small kinship coefficients with the fact that computations are made possible by this sparsity.

Thus, to enable analysis of medium-to-large genetic datasets, we implemented all computations by blocks of the kinship matrix. Because the JHS is relatively small, we artificially increased it for this analysis by (a) using a kinship matrix from all available JHS individuals in the dataset K_{jhs}, regardless of having protein measurements (n = 3,418), and (b) generating larger, blocked, kinship matrices where the blocks are K_{jhs}. We generated $3XK_{jhs}$ and $10XK_{jhs}$, with the $3X$ and $10X$ matrix being block matrices with 3 and 10 blocks, corresponding with datasets with sample sizes of 10,254 and 34,180, respectively (settings D and E).

Specific optimization steps speeding up the parametric bootstrap included the following.

(1) Simulating outcomes from a multivariate normal distribution: The parametric bootstrap requires generating random outcomes from a multivariate normal distribution corresponding to the kinship matrix. We sampled these outcomes according to the blocks of the sparse block kinship matrix, and used a function from the *mvnfast* R package, using multiple cores. The package provides computational optimization through the Rcpp\RcppArmadillo packages and parallelization through the OpenMP API. We aggregated the resulting simulated outcomes at the end of the computations.

(2) Estimation of genetic correlation and heritabilities between each pair of simulated outcomes: We implemented two custom functions using C++ combined with parallelized computations via multithreading. Thus, each of the two functions performs computations on the dataset in multiple blocks in parallel using C++ multithreading and the *Matrix* C++ class for performing mathematical operations on matrices efficiently. Results computed by each thread for separate blocks are then aggregated into the final result.

Additionally, we further reduced the memory requirements (RAM) by performing computations of genetic correlations and heritabilities in blocks of 1,000 simulations at a time (this is faster than computations of one simulation at a time).

All code is available on the open access GitHub repository https://github.com/YanaHrytsenko/Genetic_Correlations_CIs. All the computations were performed on the Amazon Web Services platform using r5a.8xlarge instances with 32 vCPUs and 256G of RAM available on each instance.

To assess the computational needs of the parametric bootstrap approach, we estimated the peak RAM use and computational time of a single parametric bootstrap computing "job" (averaged over 10 jobs). In our implementation, the parametric bootstrap (Figure 1, top) is performed in 2,000 computational jobs, according to the number of combinations of the heritability and genetic correlation parameters, each one performing 10,000 simulations. We provide these computing measures when using K_{jhs}, $3XK_{jhs}$, and $10XK_{jhs}$.

Results

Simulation studies

In primary results, for a given true value of genetic correlation ρ_k we calculated both the coverage probability and the average width of 95% CIs by averaging the corresponding estimates over all the 100 true heritability scenarios (10 for h_1^2 and 10 for h_2^2). Table S1 provides estimated coverage by all combinations of (true) ρ_k, h_1^2, h_2^2. Figure 2 provides the estimated coverage probabilities for the compared methods in simulations, and Figure 3 provides the averaged CI widths. The PMF approach provides appropriate coverage across the three settings defined by the kinship matrices. The beta approximation to the PMF results in under-coverage across the simulated ρ_k values setting A, but improved substantially in settings B and C when the simulated sample size increased. Still the average width of the CIs was lower when using the empirical PMF. In setting A, GCTA had an appropriate coverage only when ρ_k was set to 0.05. The Fisher's transformation tended to result in under-coverage.

Figure 4 compares coverage and CI widths when using the empirical PMF approach to compute CIs and settings $\rho_e = 0, 0.2,$ or 0.4 in the simulations generating data. It demonstrates that there is essentially no difference in the results.

We also used the simulations to estimate type 1 error when using the CI inversion methods with the beta approximation to the PMF to compute association p values. The results are visualized in Figure 5. Here, we also estimated type 1 error by combinations of specific h_1^2, h_2^2 values. With $\alpha = 0.05$, the type 1 error was controlled across heritability combinations in settings B and C, but not in setting A. While it is unsurprising that the type 1 error is not controlled when heritability values of either one of the two traits are very small, the test was also somewhat inflated in setting A when the two heritabilities were fairly high. Over all, the beta approximation method to the PMF is promising for computing high coverage CIs and p values when the sample size is sufficiently large.

Assessment of computing time and RAM needs

To thoroughly assess performance and computational needs in larger sample sizes, we also performed analyses of settings leveraging the kinship matrix of all JHS individuals available in the data K_{jhs}, and larger, blocked matrices, $3XK_{jhs}$, and $10XK_{jhs}$. The latter two settings ($3XK_{jhs}$ and $10XK_{jhs}$) are referred to as settings D and E, and for these we also report coverage and CI width results in Figures S1 and S2. These results demonstrate consistent patterns with settings A–C, with appropriate coverage when using both the PMF and the beta approximation to the PMF approaches, and under-coverage using the Fisher's approximation near the boundaries of the parameter space. The figures also demonstrate that the average lengths of the CIs using the beta approximation are higher than the corresponding lengths when using the PMF.

We also conducted computational performance testing on Windows 11 Pro machine, version 22H2, 64-bit, with 13th Gen Intel Core i9-13900K, 3.00 GHz, 3.72 TB of storage, 128Gb of RAM, and 32 vCPUs using *peakRAM R* package. Recall that we apply the parametric bootstrap procedures as 2,000 computational "jobs" (using computing cluster language) that perform simulations (top part in Figure 1), each with a different combination of ρ_k, h_1^2, h_2^2 parameters. Here, we report peak RAM and time elapsed for each of the major computational tasks in a computational job, and the overall time elapsed for the complete job. These estimates are averaged across 10 runs of computational job. The results are provided in Table 1. One can see that peak RAM depends on the smallest block in the blocked kinship matrix, due to the implementation of computations by blocks. The computing time of genetic correlation and heritability estimation as a part of the parametric bootstrap increases with higher overall sample sizes. Overall computing times of the parametric bootstrap computing job were 1,516, 2,428, and 11,140 s for the parametric bootstrap simulations based on K_{jhs}, $3XK_{jhs}$, and $10XK_{jhs}$, respectively.

Application to genetically determined protein-protein networks in the JHS

We estimated heritabilities and genetic correlations for every pair of proteins among the 1,317 proteins available in JHS, in an analysis adjusted to age, sex, and BMI (in which protein measures were first regressed over these covariates prior to estimation of genetic correlations based on the resulting residuals), and in an unadjusted analysis. Characteristics of the JHS dataset are provided in Table 2. Of the study participants, 61% were women. Individuals

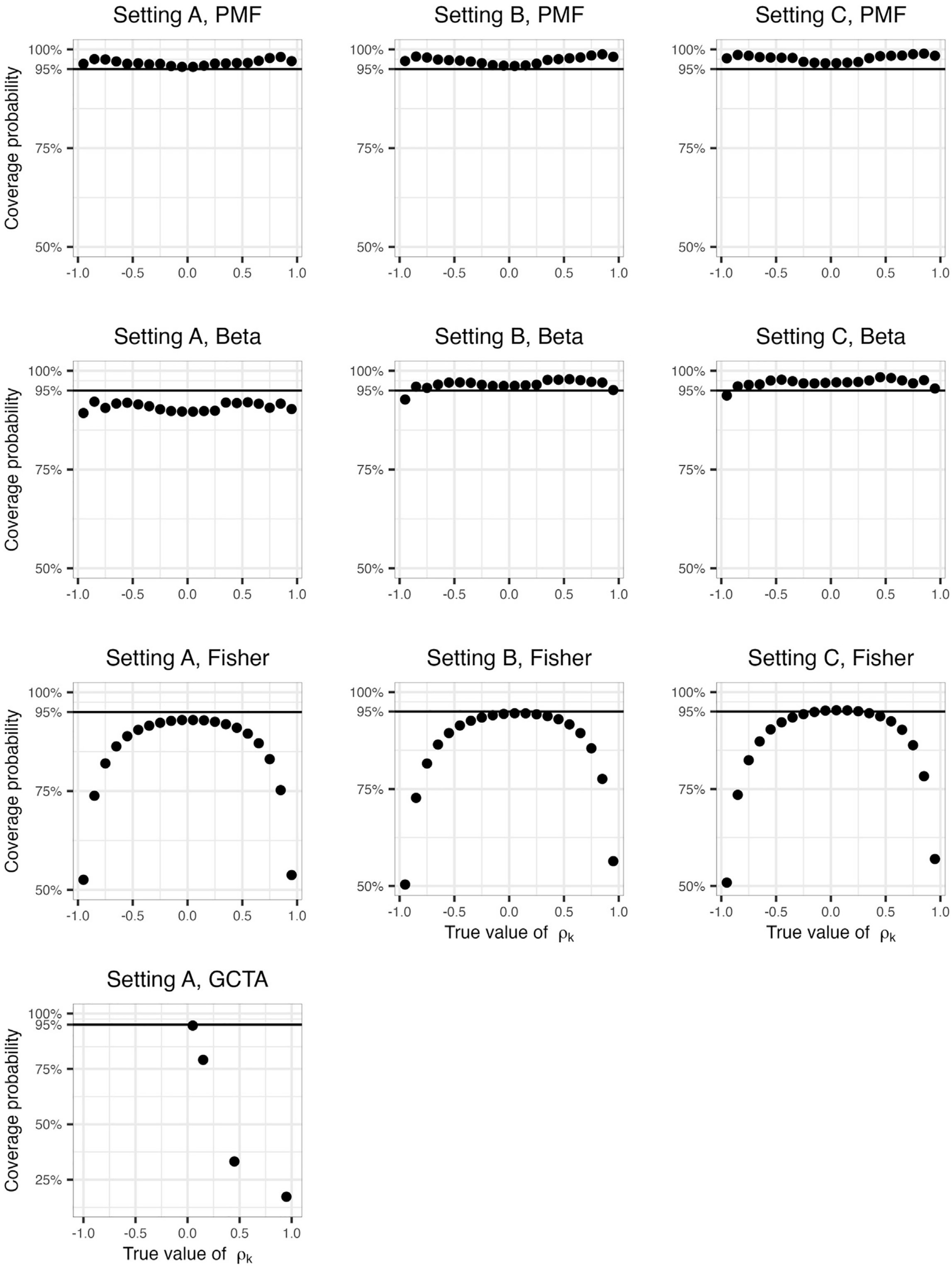

Figure 2. Estimated coverage of 95% CIs of genetic correlations in the primary simulations
The columns represent different kinship matrix sizes: Setting A denotes the use of original-size kinship matrix (n = 2,050), setting B denotes the use of the double-size kinship matrix (n = 4,100), and setting C denotes the use of the triple-size kinship matrix (n = 6,150). The rows represent the four approaches for constructing CIs, including parametric bootstrap PMF, beta approximation for parametric bootstrap PMF, Fisher's transformation, and GCTA package use of normal distribution approximation. Only parts of the analyses were carried out on the GCTA package due to the high computational resources required.

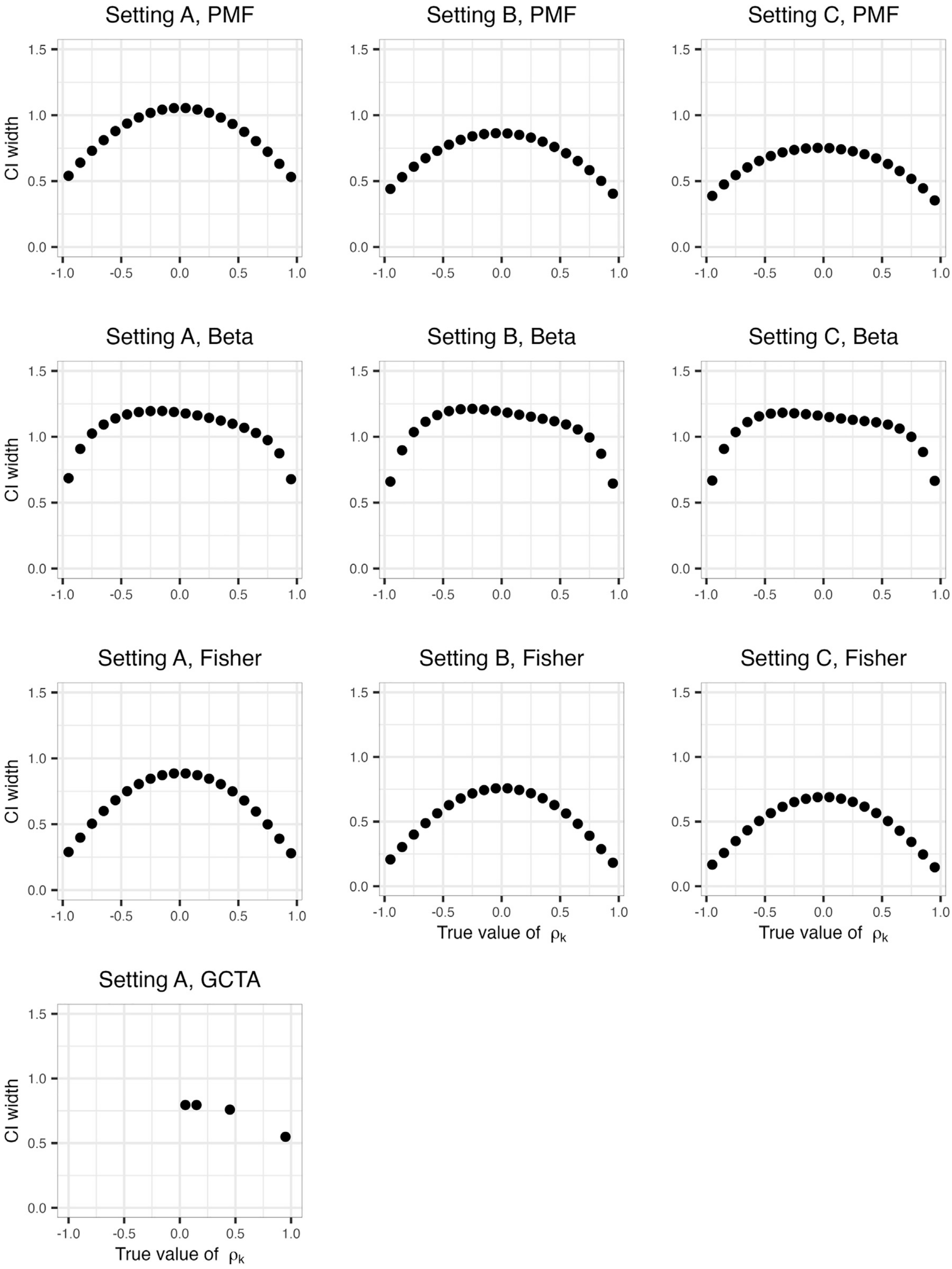

Figure 3. Average widths of the CIs of the genetic correlations in the primary simulations
The columns represent different kinship matrix sizes: Setting A denotes the use of original-size kinship matrix (n = 2,050), setting B denotes the use of the double-size kinship matrix (n = 4,100), and setting C denotes the use of the triple-size kinship matrix (n = 6,150). The rows represent the four approaches for constructing CIs, including parametric bootstrap PMF, beta approximation for parametric bootstrap PMF, Fisher's transformation, and GCTA package. Only parts of the analyses were carried out on the GCTA package due to the high computational resources required.

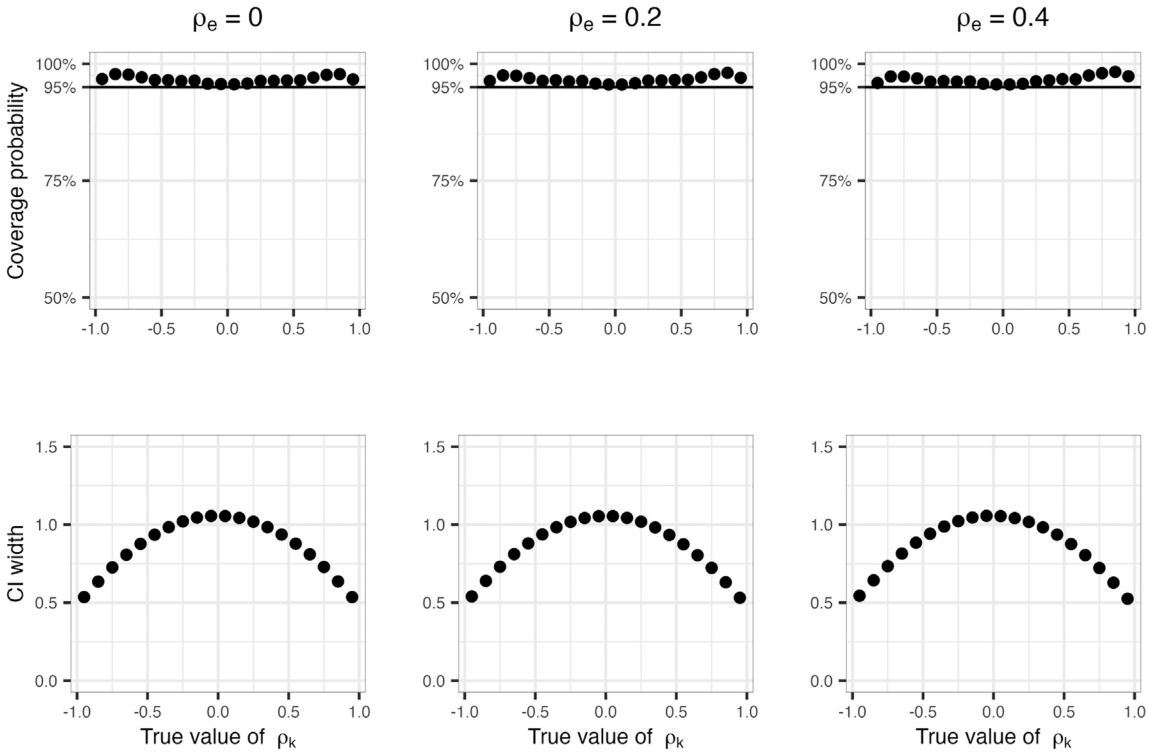

Figure 4. Setting environmental correlation between error terms ρ_e has no effect on genetic correlation estimates
Comparison of coverage and CI widths when using the empirical PMF approach to compute CIs and settings $\rho_e = 0, 0.2,$ or 0.4 in the simulations generating data.

were 55 years of age (males) and 56 years of age (females) on average, and were mostly overweight. Some individuals were close relatives. For example, there were 341 pairs of individuals with estimated coefficient of relationship $\geq$ 0.48, and 1,113 pairs of individuals with coefficient of relationship ≥ 0.12 (considering the total number of unique pairs of individuals, this corresponds with 0.05% of all pairs of participants).

Tables S2 and S3 provide the estimated heritabilities of all proteins in the dataset from analysis unadjusted and adjusted to covariates (age, sex, and BMI), respectively. Based on the simulations using this specific dataset, we removed from consideration proteins with estimated heritabilities $\widehat{h}^2 < 0.3$, as genetic correlations and p values using the beta approximation method are less reliable compared with higher values of (real, not estimated) heritabilities. We also excluded proteins with estimated $\widehat{h}^2 > 0.9$, because such a high may suggest a problem with the measurement and/or genetic characterization of a protein (e.g., technical issue with the platform, genetic variants segregated to a few families, etc.). After the above filtering, there were 403 and 431 proteins, or 81,406 and 93,096 protein-protein pairs, available for genetic correlation analysis in the covariate-adjusted and unadjusted analyses, respectively. For each set of the proteins (adjusted and unadjusted), it took approximately 2.5 h to estimate the genetic correlations and approximately 12 min to construct the CIs, based on the previously constructed parametric bootstrap reference results, for all the protein-protein pairs on a MacBook Pro laptop with an M1 chip. Full results from genetic correlation estimates for these sets of proteins are provided in Tables S4 and S5. Figure 6 visualizes the comparison between estimated phenotypic (Pearson) and genetic correlations across these phenotype pairs. The figure suggests that, for this set of highly heritable proteins, genetic correlations tend to be higher than Pearson correlations (to see this, one needs to focus in Figure 5 on the bright hexbins because they represent many more protein pairs compared to dark hexbins).

Protein-protein network

We visualize the results in a protein-protein network. Due to the large number of protein pairs, we focused the network resulting from protein-protein genetic correlations passing a p value threshold. We computed p values for the genetic correlations between the limited set of heritable and "valid" proteins (with heritability estimates that are not egregiously high) using the beta approximation to the PMF, and applied a false discovery rate (FDR) correction using the Benjamini-Hochberg procedure.[31] The considered pairs of proteins are those with FDR-adjusted genetic correlation $p < 0.01$. This corresponds with 253 and 294 pairs of genetically correlated proteins in adjusted and unadjusted analysis, respectively. Figure 7 visualizes these results. The size of each node represents its degree, with larger ones being "hub nodes/proteins," (genetically) associated with a large number of proteins. See Tables S6 and S7 for estimated genetic correlations between pairs of proteins

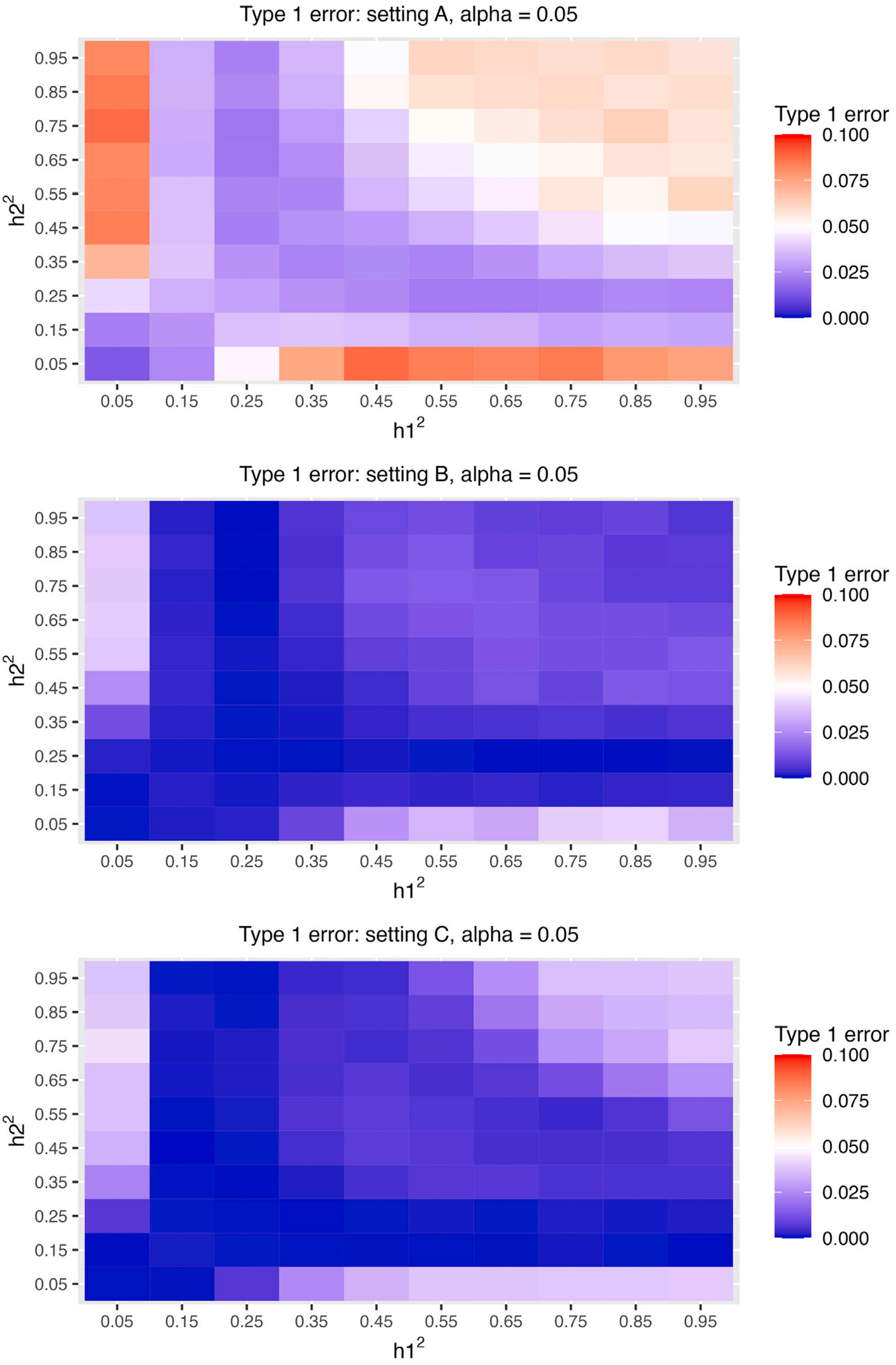

Figure 5. Type 1 error estimates when using the CI inversion method and the beta approximation to perform association testing
Visualization of type 1 error ($\rho_k = 0$) when using the CI inversion approach coupled with the beta approximation to the PMF to generate CIs. The results are provided for each simulation settings and by values of h_1^2, h_2^2.

Table 1. Peak RAM and timing analysis of parametric bootstrap computational tasks

	K_{jhs}	$3XK_{jhs}$	$10XK_{jhs}$
Load kinship matrix	RAM: 89.2<br>time elapsed: 0.15	RAM: 802.4<br>time elapsed: 0.92	RAM: 8915<br>time elapsed: 11.76
Sample 10,000 datasets from multivariate normal distribution (rmvn function)	RAM: 7,119.12<br>time elapsed: 1,317.48	RAM: 7,275.57<br>time elapsed: 1,432.53	RAM: 7,823.31<br>time elapsed: 1,514.61
Estimate genetic correlation over all 10,000 simulated outcome pairs	RAM: 14,820<br>time elapsed: 21.57	RAM: 14,820<br>time elapsed: 304.60	RAM: 3,4698.24<br>time elapsed: 3,068.57
Estimate heritability over all 10,000 simulated outcomes	RAM: 14,820<br>time elapsed: 10.10	RAM: 14,820<br>time elapsed: 149.57	RAM: 18,892.8<br>Time elapsed: 1,621.14
Overall time	time elapsed: 1,515.67	time elapsed: 2,427.97	Time elapsed: 11,139.55

The table reports computational time (seconds) and peak random access memory (RAM; MiB) for the parametric bootstrap main computational tasks in a single computing job and overall (the complete computational job). These analyses were performed using the kinship matrix of all available JHS individuals in the dataset (regardless of protein data) K_{jhs} with $n = 3{,}418$, and over block matrices mimicking larger kinship matrix $3XK_{jhs}$ with n = 10,254, and $10XK_{jhs}$ with n = 34,180. Load kinship matrix refers to reading a full-sized kinship matrix saved as an R RDS file. Note that the heritability estimation step is performed twice: once for each simulated outcome out of the pair of outcomes.

selected based on the criteria described above. Table S8 contains a list of the top 10 hub nodes/proteins and their connections, i.e., the list of proteins connected to each of these hub nodes, both in covariate-adjusted and unadjusted analyses. Visually, the network seems to be less connected (and we also know that the number of connections decreased) in analysis that adjusted for age, sex, and BMI. It is likely that genetic correlations decreased because BMI has strong effects on proteins, and the genetic effects on BMI are also strong, so when BMI was adjusted for, genetic effects inducing correlations between proteins were reduced.

Discussion

We developed a parametric bootstrap procedure to estimate CIs for the genetic correlation estimator, studied it in simulations, and applied it to learn a protein-protein network using a set of heritable proteins measured in the JHS. The proposed procedure outperforms existing methods in that the resulting CI are not under-covered across the range of potential genetic correlation parameters.

Our bootstrap procedure was inspired by a similar approach developed for heritability CIs.[19] Compared with the previous publication focusing on heritability, our approach is complicated by the need to simulate pairs of traits, including their heritabilities and genetic correlation between them, i.e., a grid of three parameters rather than one. Indeed, CIs for genetic correlation depend on trait heritability and are wider when at least one of the traits has low heritability. Thus, the computation burden of our procedure is higher. It is especially important to recognize that this procedure, like that of Schweiger et al.,[19] is dataset dependent, because it uses the kinship matrix of the specific dataset. However, our procedure is realistic and useful when many genetic correlations are estimated for the same dataset, as in this work. In this case, the parametric bootstrap simulation step is performed once, but is applied many times. A limitation posed by the high dimensional number of parameters required by the bootstrap procedure (many genetic correlation parameters) is the limited level of coverage due to the discreteness of the bootstrap procedure: we cannot use the estimated conditional PMF of ρ_k (conditional on the estimated genetic correlation and heritabilities), as it is to obtain CIs at the 1-α level when α is very small (e.g., 10^{-7}). To address this, we proposed the beta approximation, after local-scale transformation, to the PMF. The beta distribution has two parameters that can be fit to approximate many distribution functions that are on a bounded interval. Based on our simulations, CIs based on the beta approximation tend to be wider than those using the PMF directly, and they can still under-cover the desired distribution in low sample sizes. However, for larger sample sizes their performance improves. Overall, we think that for larger sample sizes, e.g., 6,000 individuals, the beta approximation to the PMF will be very useful in providing reliable CIs and, using the inversion method, p values. It is important to point out that while we performed simulations with a "triple size" JHS kinship matrix, i.e., of n = 6,150 individuals, the effective sample size corresponding with it is much lower than that of real potential datasets with 6,150 individuals. That is, because we simulated a block diagonal matrix. Realistic kinship matrices will have non-zero off diagonal values throughout (unless forced to be zero for computational efficiency purposes[30]).

We implemented a binary search procedure to compute p values, via the computation of CIs of varying degrees of coverage, i.e., the binary search is on the α level of the coverage parameter—where the binary search stops when the maximum coverage that does not include 0 is attained (and once we see that the next potential level of coverage results in a CI that includes 0). Binary search is useful when (a) the function is monotone (as the α value decreases eventually we will "yes" cover zero, for all smaller α), and (b) the function cannot be transformed: we cannot create a function that takes as an input "yes" or "no" and output an α value. The binary search uses a sensitivity parameter, which may be determined by the desired α level. For example, if a researcher prespecifies a criterion for "significance" of

Table 2. JHS dataset characteristics stratified by sex

Characteristic	Female	Male
N	1,252	798
Age, years[a]	57 (46–65)	55 (45–65)
BMI[a]	32 (27–37)	29 (26–32)

[a]Median (interquartile range).

genetic correlation as $p < 1 \times 10^{-5}$, it is important to be able to determine whether a CI value when $\alpha = 1 \times 10^{-5}$ covers zero, and so the sensitivity level should be no higher than 1×10^{-5}; otherwise, associations with p values close to this value may not unambiguously be identified as statistically significant. Thus, the sensitivity level affects power and type 1 error only in the sense that one could determine that a p value is lower than a pre-specified level, but no lower (e.g., one could determine that a $p < \alpha$ or that but not that $p = \alpha/c$ for a $c > 1$). Here, we note that another limitation to the attain sensitivity is that the PMF computation is based on a limited number of simulations, again resulting in a discreate function and thus limited coverage/p value sensitivity. The beta approximation to the PMF addresses this limitation, because the beta function is continuous and, therefore, does not have limited sensitivity.

Existing methods that compute CIs for genetic correlations typically utilize an asymptotic normal distribution argument, at either the untransformed or Fisher's transformation level. This is appropriate depending on the combination of four factors: sample size, underlying (true) heritabilities of each of the pair of traits, and the underlying genetic correlation. For any given pair of traits and a dataset, any one of these factors may be suboptimal, potentially leading to poor performance of CIs that rely on asymptotic normality. The bootstrap procedure addresses this shortcoming. However, this procedure too does not produce perfect CIs: for low values of heritability of either one of the two traits, the coverage may still be lower than desired in low sample sizes. Note that in reality we do not know the true heritability, we only have estimated heritability. Therefore, we cannot tell whether a CI may not be reliable according to the values of the estimated heritabilities. That is why our main results are provided at an aggregate level, across simulated values of potential heritabilities.

We demonstrated the use of genetic correlations to infer genetically determined protein-protein networks. However, we acknowledge that our analysis is limited by the relatively low sample size, which led to posing a stringent filter requiring at least 0.3 estimated protein heritability for inclusion in the downstream analysis. While we chose to include only edges with estimated FDR-adjusted $p < 0.01$ (with p values estimated using the beta approximation), other statistical network approaches may generate sparsity using penalized multivariant regression techniques.[32,33] It would be interesting to extend such approaches to genetic (rather than phenotypic) correlation-based networks. In future work, we will apply the existing framework on larger datasets and develop approximation methods to further speed up the simulations required for the parametric bootstrap and the estimation of heritabilities and genetic correlations, for example, following Wu et al.[34]

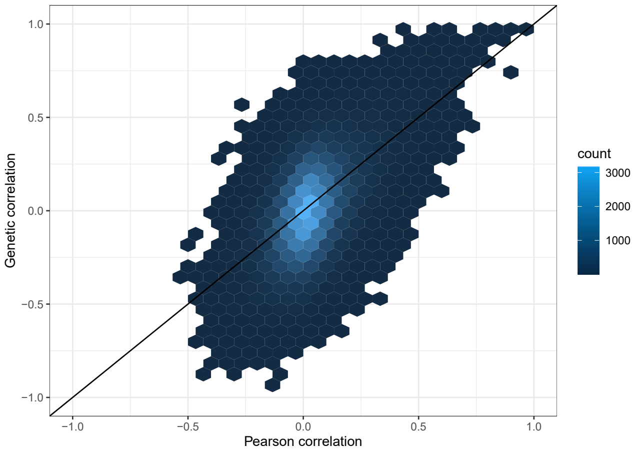

Figure 6. Estimated Pearson versus genetic correlations between heritable proteins
The figure compares the sample Pearson correlation with the estimated genetic correlation $\hat{\rho}_k$ for all protein pairs for which the estimated heritability $\hat{h}^2 > 0.3$ for each of the proteins. The color of each hexbin represents the number (count) of protein pairs with x- and y axis values falling under the hexbin.

Data and code availability

Code to implement the parametric bootstrap approach is provided in the public GitHub repository https://github.com/YanaHrytsenko/Genetic_Correlations_CIs. Genetic data from the JHS are available via a data use agreement with the database of genotypes and phenotypes (dbGaP), study accession "dbGaP: phs000286". WGS data are available via a data use agreement with dbGaP project "NHLBI TOPMed: The Jackson Heart Study (JHS)," study accession "dbGaP: phs000964". The JHS proteomics dataset used in this analysis is available via a data use agreement with the JHS Data Coordinating Center (DCC), see website https://www.jacksonheartstudy.org/. Alternatively, JHS DCC can be contacted via the email jhsccdc@umc.edu.

Supplemental information

Supplemental information can be found online at https://doi.org/10.1016/j.xhgg.2024.100304.

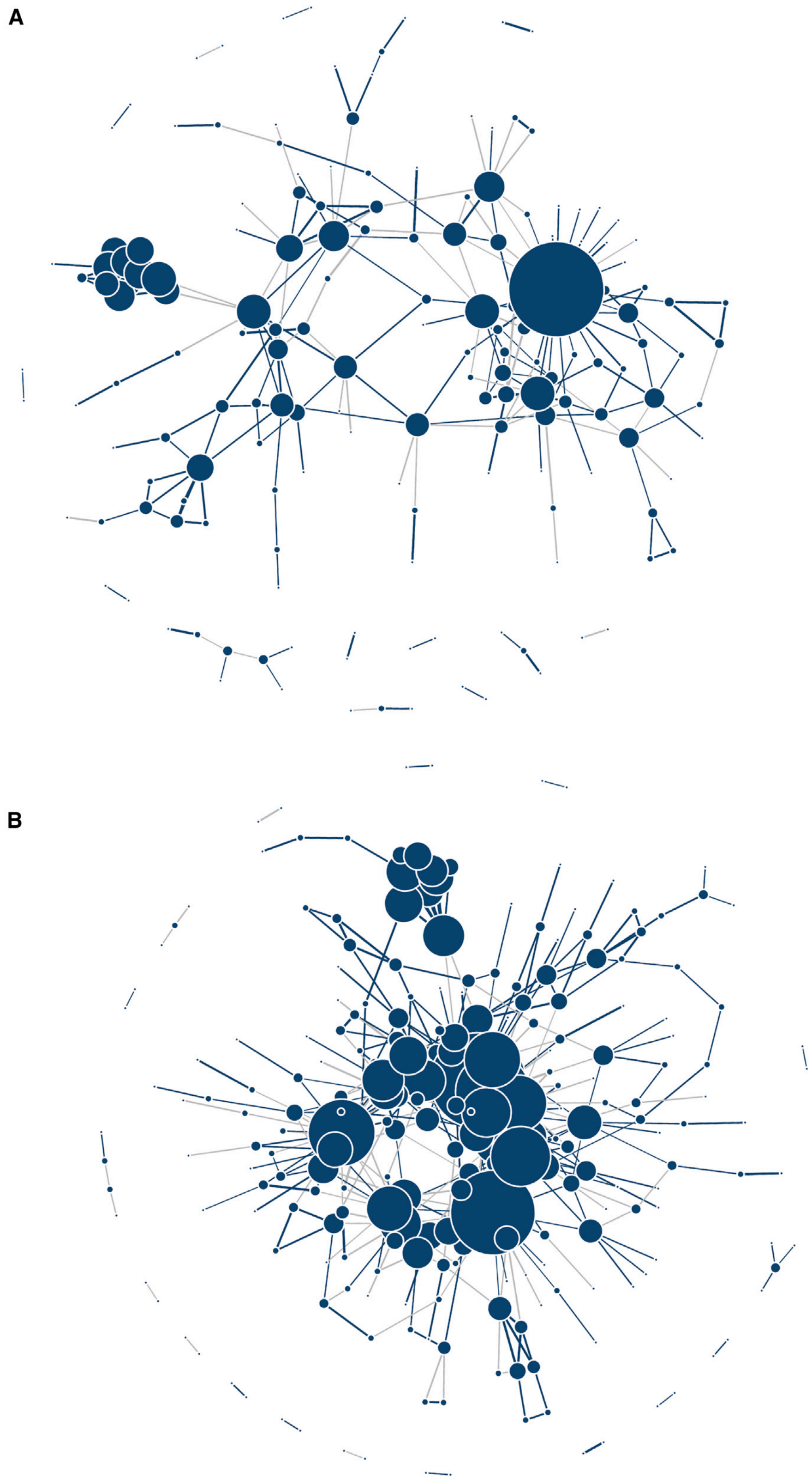

Figure 7. Networks constructed from top pairs of genetically correlated proteins
(A) Protein-protein genetic correlation network using the age, sex, BMI-adjusted proteins. (B) The corresponding network based on unadjusted analysis. The blue edges represent positive genetic correlations, and the gray edges represent negative genetic correlations. Larger nodes are hub proteins where multiple proteins have strong genetic correlations with each other both in covariate adjusted and unadjusted analyses. Some of the hub proteins include *APO_D*, *PREKALLIKREIN*, *NOTCH_3*, *HPV_E7_TYPE18*, *CARBONIC_ANHYDRASE_IV*, *CDK5_P35*, *DKK_4*, *PAK3*, *TRKC*, *MIS*, *C5A*, *OMD*, *JAG1*, *HEPARIN_COFACTOR_II*, *BFGF_R*, *and MMP_2*, and *GDF_11_8*.

Acknowledgments

This work was supported by the National Institute of Diabetes and Digestive and Kidney Diseases R01DK081572. The Jackson Heart Study (JHS) is supported and conducted in collaboration with Jackson State University (HHSN268201800013I), Tougaloo College (HHSN268201800014I), the Mississippi State Department of Health (HHSN268201800015I), and the University of Mississippi Medical Center (HHSN268201800010I, HHSN268201800011I, and HHSN268201800012I) contracts from the National Heart, Lung, and Blood Institute (NHLBI) and the National Institute on Minority Health and Health Disparities (NIMHD). The authors also wish to thank the staffs and participants of the JHS. The views expressed in this manuscript are those of the authors and do not necessarily represent the views of the National Heart, Lung, and Blood Institute; the National Institutes of Health; or the U.S. Department of Health and Human Services.

Author contributions

Y.-T.T., Y.H., M.E., and T.S. developed the method and the code. J.G.W., R.E.G. collected the data. Y.-T.T. and Y.H. performed data analysis and visualization. U.A.T., Z.-Z.C., J.G.W., and R.E.G. reviewed and interpreted results from data analysis. Y.-T.T., Y.H., and T.S. drafted the original manuscript. M.E., U.A.T., Z.-Z.C., J.G.W., and R.E.G., critically reviewed the manuscript.

Declaration of interests

The authors declare no competing interests.

Received: October 30, 2023
Accepted: May 4, 2024

References

1. van Rheenen, W., Peyrot, W.J., Schork, A.J., Lee, S.H., and Wray, N.R. (2019). Genetic correlations of polygenic disease traits: from theory to practice. Nat. Rev. Genet. *20*, 567–581.
2. Visscher, P.M., Hill, W.G., and Wray, N.R. (2008). Heritability in the genomics era–concepts and misconceptions. Nat. Rev. Genet. *9*, 255–266.
3. Bulik-Sullivan, B., Finucane, H.K., Anttila, V., Gusev, A., Day, F.R., Loh, P.-R., ReproGen Consortium; Psychiatric Genomics Consortium; and Genetic Consortium for Anorexia Nervosa of the Wellcome Trust Case Control Consortium 3, and Duncan, L., et al. (2015). An atlas of genetic correlations across human diseases and traits. Nat. Genet. *47*, 1236–1241.
4. O'Connor, L.J., and Price, A.L. (2018). Distinguishing genetic correlation from causation across 52 diseases and complex traits. Nat. Genet. *50*, 1728–1734.
5. Zhang, Y., Elgart, M., Kurniansyah, N., Spitzer, B.W., Wang, H., Kim, D., Shah, N., Daviglus, M., Zee, P.C., Cai, J., et al. (2022). Genetic determinants of cardiometabolic and pulmonary phenotypes and obstructive sleep apnoea in HCHS/SOL. EBioMedicine *84*, 104288.
6. Ikeda, M., Tanaka, S., Saito, T., Ozaki, N., Kamatani, Y., and Iwata, N. (2018). Re-evaluating classical body type theories: genetic correlation between psychiatric disorders and body mass index. Psychol. Med. *48*, 1745–1748.
7. Kappelmann, N., Arloth, J., Georgakis, M.K., Czamara, D., Rost, N., Ligthart, S., Khandaker, G.M., and Binder, E.B. (2021). Dissecting the Association Between Inflammation, Metabolic Dysregulation, and Specific Depressive Symptoms: A Genetic Correlation and 2-Sample Mendelian Randomization Study. JAMA Psychiatr. *78*, 161–170.
8. Shi, H., Mancuso, N., Spendlove, S., and Pasaniuc, B. (2017). Local Genetic Correlation Gives Insights into the Shared Genetic Architecture of Complex Traits. Am. J. Hum. Genet. *101*, 737–751.
9. Zhang, Y., Lu, Q., Ye, Y., Huang, K., Liu, W., Wu, Y., Zhong, X., Li, B., Yu, Z., Travers, B.G., et al. (2021). SUPERGNOVA: local genetic correlation analysis reveals heterogeneous etiologic sharing of complex traits. Genome Biol. *22*, 262.
10. Guo, H., Li, J.J., Lu, Q., and Hou, L. (2021). Detecting local genetic correlations with scan statistics. Nat. Commun. *12*, 2033.
11. Werme, J., van der Sluis, S., Posthuma, D., and de Leeuw, C.A. (2022). An integrated framework for local genetic correlation analysis. Nat. Genet. *54*, 274–282.
12. Lu, Q., Li, B., Ou, D., Erlendsdottir, M., Powles, R.L., Jiang, T., Hu, Y., Chang, D., Jin, C., Dai, W., et al. (2017). A Powerful Approach to Estimating Annotation-Stratified Genetic Covariance via GWAS Summary Statistics. Am. J. Hum. Genet. *101*, 939–964.
13. Weissbrod, O., Flint, J., and Rosset, S. (2018). Estimating SNP-Based Heritability and Genetic Correlation in Case-Control Studies Directly and with Summary Statistics. Am. J. Hum. Genet. *103*, 89–99.
14. Zhang, Y., Cheng, Y., Jiang, W., Ye, Y., Lu, Q., and Zhao, H. (2021). Comparison of methods for estimating genetic correlation between complex traits using GWAS summary statistics. Brief. Bioinform. *22*, bbaa442.
15. Elgart, M., Goodman, M.O., Isasi, C., Chen, H., Morrison, A.C., de Vries, P.S., Xu, H., Manichaikul, A.W., Guo, X., Franceschini, N., et al. (2022). Correlations between complex human phenotypes vary by genetic background, gender, and environment. Cell Rep. Med. *3*, 100844.
16. Furlotte, N.A., and Eskin, E. (2015). Efficient multiple-trait association and estimation of genetic correlation using the matrix-variate linear mixed model. Genetics *200*, 59–68.
17. Lee, S.H., and van der Werf, J.H.J. (2016). MTG2: an efficient algorithm for multivariate linear mixed model analysis based on genomic information. Bioinformatics *32*, 1420–1422.
18. Lee, S.H., Yang, J., Goddard, M.E., Visscher, P.M., and Wray, N.R. (2012). Estimation of pleiotropy between complex diseases using single-nucleotide polymorphism-derived genomic relationships and restricted maximum likelihood. Bioinformatics *28*, 2540–2542.
19. Schweiger, R., Kaufman, S., Laaksonen, R., Kleber, M.E., März, W., Eskin, E., Rosset, S., and Halperin, E. (2016). Fast and accurate construction of confidence intervals for heritability. Am. J. Hum. Genet. *98*, 1181–1192.
20. Sofer, T. (2017). Confidence intervals for heritability via Haseman-Elston regression. Stat. Appl. Genet. Mol. Biol. *16*, 259–273.
21. Brown, G.H. (1969). An empirical study of the distribution of the sample genetic correlation coefficient. Biometrics *25*, 63–72.
22. Balding, D.J. (2003). Likelihood-based inference for genetic correlation coefficients. Theor. Popul. Biol. *63*, 221–230.
23. Liu, B.H., Knapp, S.J., and Birkes, D. (1997). Sampling distributions, biases, variances, and confidence intervals for genetic correlations. Theor. Appl. Genet. *94*, 8–19.
24. Legarra, A. (2016). Comparing estimates of genetic variance across different relationship models. Theor. Popul. Biol. *107*, 26–30.
25. Wyatt, S.B., Diekelmann, N., Henderson, F., Andrew, M.E., Billingsley, G., Felder, S.H., Fuqua, S., and Jackson, P.B. (2003). A community-driven model of research participation:

the Jackson Heart Study Participant Recruitment and Retention Study. Ethn. Dis. *13*, 438–455.

26. Taylor, H.A., Wilson, J.G., Jones, D.W., Sarpong, D.F., Srinivasan, A., Garrison, R.J., Nelson, C., and Wyatt, S.B. (2005). Toward resolution of cardiovascular health disparities in African Americans: design and methods of the Jackson Heart Study. Ethn. Dis. *15*, S6–S17.
27. Katz, D.H., Tahir, U.A., Bick, A.G., Pampana, A., Ngo, D., Benson, M.D., Yu, Z., Robbins, J.M., Chen, Z.Z., Cruz, D.E., et al. (2022). Whole genome sequence analysis of the plasma proteome in black adults provides novel insights into cardiovascular disease. Circulation *145*, 357–370.
28. Yang, J., Lee, S.H., Goddard, M.E., and Visscher, P.M. (2011). GCTA: a tool for genome-wide complex trait analysis. Am. J. Hum. Genet. *88*, 76–82.
29. Mudholkar, G.S., and Chaubey, Y.P. (1976). On the distribution of Fisher's transformation of the correlation coefficient. Commun. Stat. Simulat. Comput. *5*, 163–172.
30. Gogarten, S.M., Sofer, T., Chen, H., Yu, C., Brody, J.A., Thornton, T.A., Rice, K.M., and Conomos, M.P. (2019). Genetic association testing using the GENESIS R/Bioconductor package. Bioinformatics *35*, 5346–5348.
31. Benjamini, Y., and Hochberg, Y. (1995). Controlling the false discovery rate: a practical and powerful approach to multiple testing. J. Roy. Stat. Soc. B *57*, 289–300.
32. Li, H., and Gui, J. (2006). Gradient directed regularization for sparse Gaussian concentration graphs, with applications to inference of genetic networks. Biostatistics *7*, 302–317.
33. Li, B., Chuns, H., and Zhao, H. (2012). Sparse estimation of conditional graphical models with application to gene networks. J. Am. Stat. Assoc. *107*, 152–167.
34. Wu, Y., Burch, K.S., Ganna, A., Pajukanta, P., Pasaniuc, B., and Sankararaman, S. (2022). Fast estimation of genetic correlation for biobank-scale data. Am. J. Hum. Genet. *109*, 24–32.

HGGA, Volume *5*

Supplemental information

A parametric bootstrap approach for computing confidence intervals for genetic correlations with application to genetically determined protein-protein networks

Yi-Ting Tsai, Yana Hrytsenko, Michael Elgart, Usman A. Tahir, Zsu-Zsu Chen, James G. Wilson, Robert E. Gerszten, and Tamar Sofer

A parametric bootstrap approach for computing confidence intervals for genetic correlations with application to genetically-determined protein-protein networks: Supplementary Note

Decomposition of the total phenotypic variance into genetic and residual variance. 1
Derivation of the two outcomes covariance model. 2

Supplementary Note

Decomposition of the total phenotypic variance into genetic and residual variance.

Suppose that the linear model relating the outcome vector $\boldsymbol{y}$ (of length $n \times 1$) to covariates $\boldsymbol{X}$ and genetic effects $\boldsymbol{G}$ is given by

$$\boldsymbol{y} = \boldsymbol{X\beta} + \boldsymbol{G\alpha} + \boldsymbol{e},$$

Where $\boldsymbol{e}$ is a vector of errors independent of the genetic effects. Conditional on covariates $\boldsymbol{X}$, the covariance matrix of $\boldsymbol{y}$ can be written as:

$$cov(\boldsymbol{y} - \boldsymbol{X\beta}, \boldsymbol{y} - \boldsymbol{X\beta}) = cov(\boldsymbol{G\alpha} + \boldsymbol{e}, \boldsymbol{G\alpha} + \boldsymbol{e}) = cov(\boldsymbol{G\alpha}) + cov(\boldsymbol{e})$$

Where $cov(\boldsymbol{e}) = \sigma_e^2 \boldsymbol{I}_{n\times n}$, such that the errors are assumed independent across individuals.

To consider the genetic covariance between individuals, first let's assume that there are d variants modelled, and under approximating assumptions of independence of genetic variants, standardized to have mean 0 and variance 1. Then, for individual i:

$$cov(\boldsymbol{g}_i^T \boldsymbol{\alpha}) = var(\boldsymbol{g}_i^T \boldsymbol{\alpha}) = var(g_{i1}\alpha_1 + \cdots + g_{id}\alpha_d) =$$

$$= var(g_{i1})\alpha_1^2 + \cdots + var(g_{id})\alpha_d^2$$

$$= \sum_{j=1}^{d} \alpha_j^2 = \sigma_k^2$$

Where σ_k^2 is the genetic variance.

To model the covariance between the outcome of the i and j individuals, we rely on the over-all probability (i.e., across the genome) that a variant is identical-by-descent between the two individuals. For this we use the kinship coefficient k_{ij}, and have:

$$cov\left(\boldsymbol{g}_i^T \boldsymbol{\alpha}, \boldsymbol{g}_j^T \boldsymbol{\alpha}\right) = cov\left(g_{i1}\alpha_1 + \cdots + g_{id}\alpha_d, g_{j1}\alpha_1 + \cdots + g_{jd}\alpha_d\right) =$$

$$= cov\left(g_{i1}, g_{j1}\right)\alpha_1^2 + \cdots + cov\left(g_{id}, g_{jd}\right)\alpha_d^2$$
$$= k_{ij}\sum_{j=1}^{d}\alpha_j^2 = k_{ij}\sigma_k^2$$

Note here that

$$\sum_{j=1}^{d}\alpha_j^2 = \sigma_k^2$$

Can also be formulated as the variance of genetic effects, when the vector of genetic effects is viewed as a vector of fandom variable drawn from the same distribution with mean 0.

Combining everything, we get that $cov(\boldsymbol{y}|\boldsymbol{X\beta}) = \sigma_k^2\boldsymbol{K} + \sigma_e^2\boldsymbol{I}_n$.

Derivation of the two outcomes covariance model.

In the main manuscript, we stated that given two $n \times 1$ vectors $\boldsymbol{y_1}, \boldsymbol{y_2}$, their covariance can be modelled as

$$cov(\boldsymbol{y_1},\ \boldsymbol{y_2}) = \sigma_{k,1}\sigma_{k,2}\ \rho_k\ \boldsymbol{K} + \sigma_{e,1}\sigma_{e,2}\rho_e\boldsymbol{I}_n,$$

Where $\boldsymbol{K}$ is the kinship matrix and $\boldsymbol{I}_n = I_{n\times n}$ is the $n \times n$ identity matrix with diagonal values equal to 1 and the rest of the values being equal to zero.

To explain this model, note that $cov(\boldsymbol{y_1},\ \boldsymbol{y_2})$ is an $n \times n$ matrix, where the i, j entry provides the covariance $cov\left(y_{i1}, y_{j2}\right)$ where y_{i1} is outcome 1 of individua i, and y_{j2} is outcome 2 of individual j.

To demonstrate the model, we return to the definition that uses the genetic effects, as in the previous section. Let the vector of genetic effects of outcomes y_1 and y_2 be $\boldsymbol{\alpha}_1$ and $\boldsymbol{\alpha}_2$, respectively.

When $i = j$, i.e. the two outcomes are of the same person, because $k_{ii} = 1$,

$$cov(\boldsymbol{g}_i^T\boldsymbol{\alpha}_1 + e_{i1}, \boldsymbol{g}_i^T\boldsymbol{\alpha}_2 + e_{i2})$$
$$= cov\left(g_{i1}\alpha_{11} + \cdots + g_{id}\alpha_{d1} + e_{i1}, g_{j1}\alpha_{12} + \cdots + g_{jd}\alpha_{d2} + e_{i2}\right) =$$
$$= cov\left(g_{i1}, g_{j1}\right)\alpha_{11}\alpha_{12} + \cdots + cov\left(g_{id}, g_{jd}\right)\alpha_{21}\alpha_{22} + cov(e_{i1}, e_{i2}) =$$
$$= \sum_{j=1}^{d}\alpha_{j1}\alpha_{j2} + cov(e_{i1}, e_{i2})$$

Treating the vector of variant effect sizes $\boldsymbol{\alpha}_1$ and $\boldsymbol{\alpha}_2$ as vectors of random variables with mean 0, we see that the sum

$$\sum_{j=1}^{d} \alpha_{j1}\alpha_{j2}$$

Expresses the covariance between the elements in the vectors. Covariance of two random variables x_1 and x_2 is defined as: $cov(x_1, x_2) = cor(x_1, x_2) \times \sqrt{var(x_1)var(x_2)}$.
From here follows the model where

$$\sum_{j=1}^{d} \alpha_{j1}\alpha_{j2} = cor(\alpha_1, \alpha_2) \times \sigma_{k1}\sigma_{k2} = \rho_k \sigma_{k1}\sigma_{k2}$$

Where σ_{k1} is defined as the variance of the entries of the $\boldsymbol{\alpha}_1$ vector as before, similarly for α_{k2}, and ρ_k is the genetic correlation between y_1, y_2, formulated as the correlation between the set of genetic effects of the two outcomes.

Similarly,

$$cov(e_{i1}, e_{i2}) = cor(e_{i1}, e_{i2}) = cor(e_1, e_2) \times \sigma_{e1}\sigma_{e2} = \rho_e \sigma_{e1}\sigma_{e2}.$$

When $i \neq j$, the derivation is similar. Notable differences are that e_{i1} is independent of e_{j2}, so that $cov(e_{i1}, e_{j2}) = 0$, and that $cov(g_{il}, g_{jl}) = k_{ij}$ rather than 1, for $l = 1, \dots, d$.

$$\begin{aligned} &cov(\boldsymbol{g}_i^T \boldsymbol{\alpha}_1 + e_{i1}, \boldsymbol{g}_j^T \boldsymbol{\alpha}_2 + e_{j2}) \\ &\qquad\qquad = cov(g_{i1}\alpha_{11} + \dots + g_{id}\alpha_{d1} + e_{i1}, g_{j1}\alpha_{12} + \dots + g_{jd}\alpha_{d2} + e_{j2}) = \\ &= cov(g_{i1}, g_{j1})\alpha_{11}\alpha_{12} + \dots + cov(g_{id}, g_{jd})\alpha_{21}\alpha_{22} + cov(e_{i1}, e_{j2}) = \\ &= k_{ij} \sum_{j=1}^{d} \alpha_{j1}\alpha_{j2} \end{aligned}$$

Therefore:

$$cov(\boldsymbol{g}_i^T \boldsymbol{\alpha}_1 + e_{i1}, \boldsymbol{g}_j^T \boldsymbol{\alpha}_2 + e_{j2}) = k_{ij}\rho_k \sigma_{k1}\sigma_{k2}$$

Organizing all the elements in the matrix $cov(\boldsymbol{y_1}, \boldsymbol{y_2})$, we get that

$$cov(\boldsymbol{y_1}, \boldsymbol{y_2}) = \sigma_{k,1}\sigma_{k,2}\, \rho_k\, \boldsymbol{K} + \sigma_{e,1}\sigma_{e,2}\rho_e \boldsymbol{I_n}.$$

Supplementary Figures

Figure S1: Estimated coverage probabilities in Settings D and E

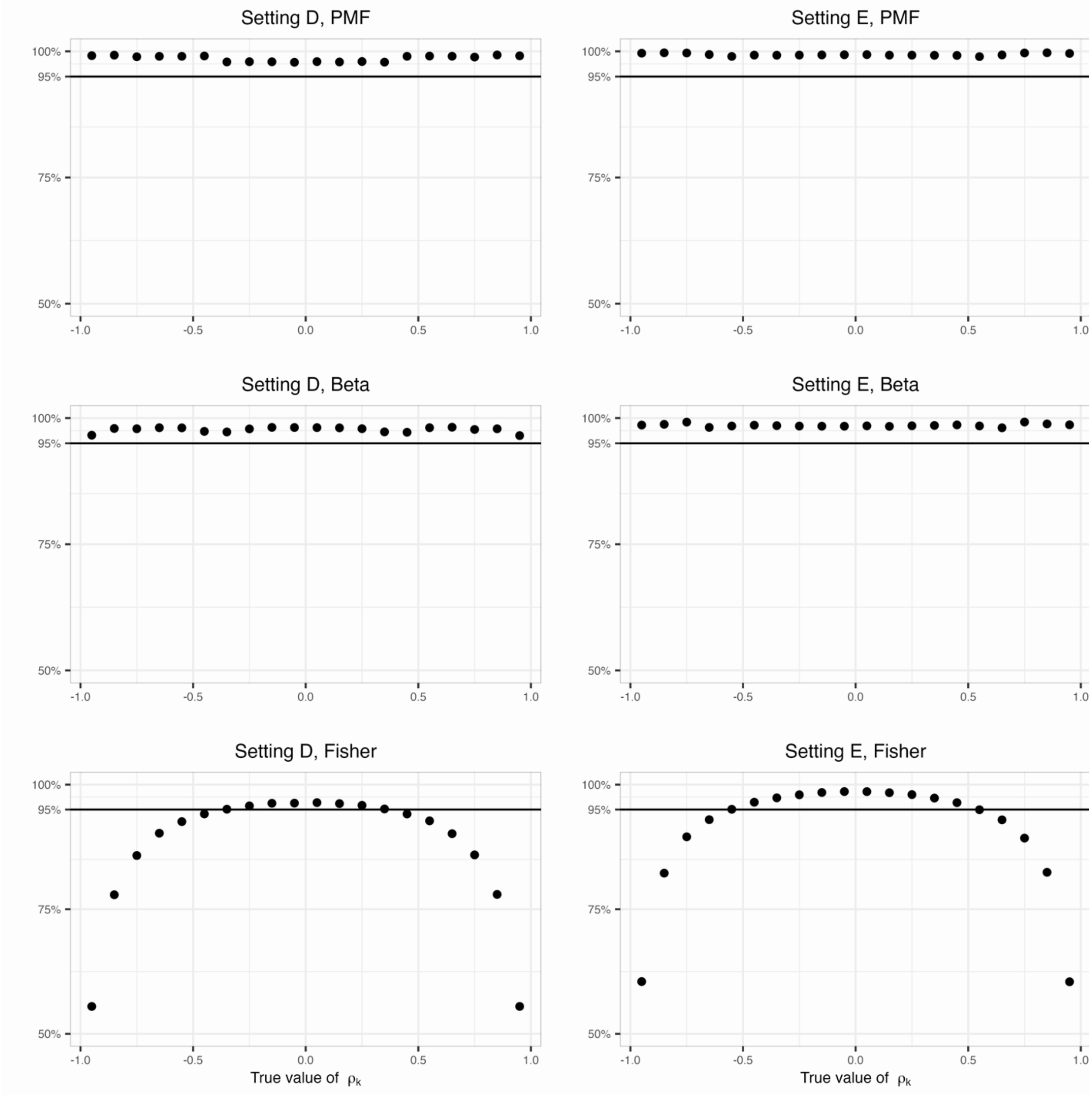

The columns represent different kinship matrix sizes: Setting D denotes the use of the block matrix with three blocks, each in the size all JHS kinship matrix ($3XK_{jhs}$, n=10,254), and Setting E denotes the use of the 10-size kinship matrix ($10XK_{jhs}$, n=34,180). The rows represent three approaches for constructing CIs, including parametric bootstrap PMF, beta approximation for parametric bootstrap PMF, and Fisher's transformation.

Figure S2: Mean CI lengths in Settings D and E

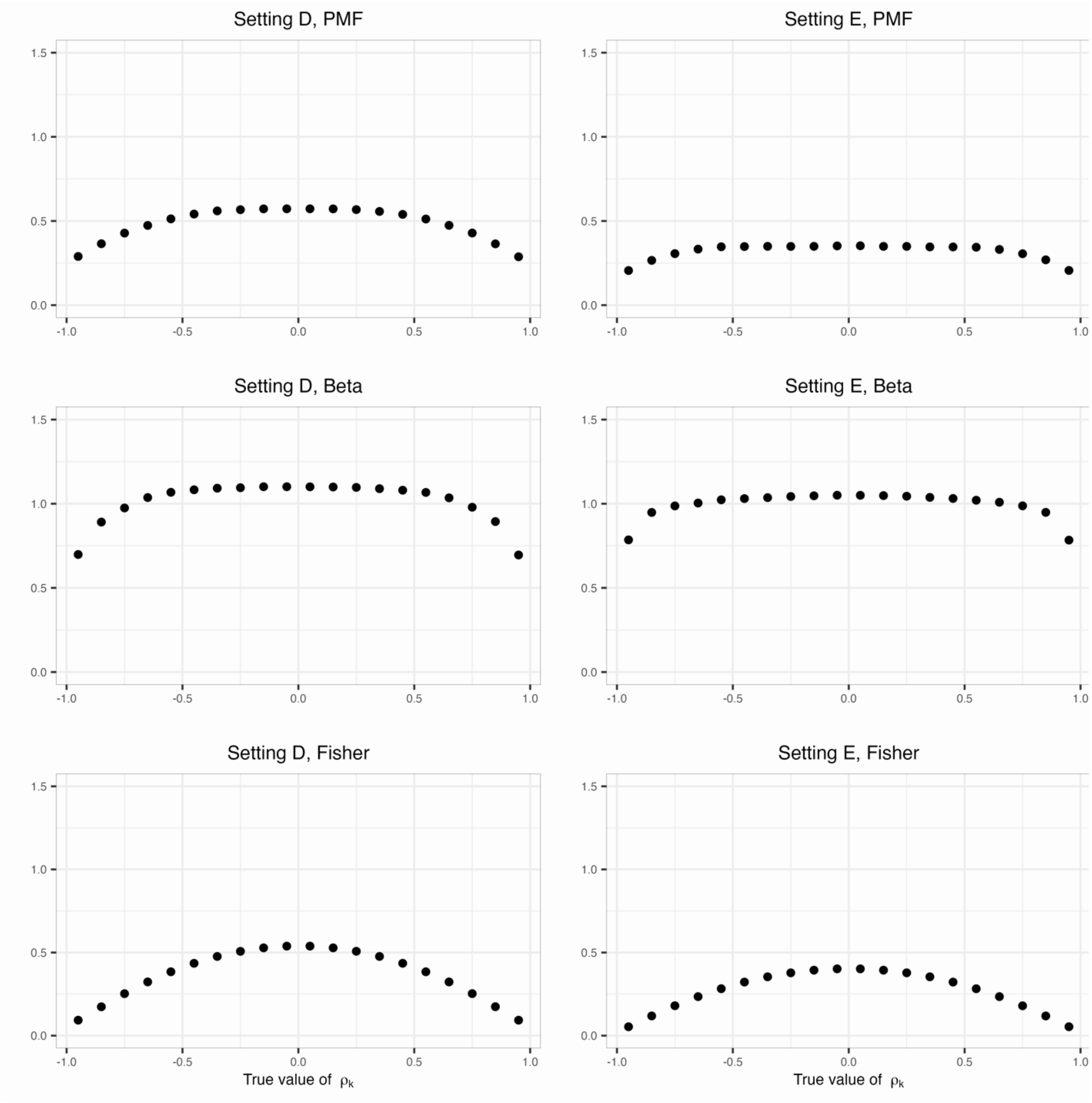

The columns represent different kinship matrix sizes: Setting D denotes the use of the block matrix with three blocks, each in the size all JHS kinship matrix ($3XK_{jhs}$, n=10,254), and Setting E denotes the use of the 10-size kinship matrix ($10XK_{jhs}$, n=34,180). The rows represent three approaches for constructing CIs, including parametric bootstrap PMF, beta approximation for parametric bootstrap PMF, and Fisher's transformation.
